# Supplementary material for: Visualizing intraorganellar ultrastructures, dynamics, and interactions with open-access background-free Lock-in-SIM
Source: Nat Commun. 2025 Nov 28;16:10765. doi: 10.1038/s41467-025-65805-w (PMC12663579; doi:10.1038/s41467-025-65805-w)
Supplement: Supplementary file 1 — Supplementary Information [file 41467_2025_65805_MOESM1_ESM.pdf]

SUPPLEMENTARY INFORMATION

Visualizing intraorganellar ultrastructures, dynamics, and interactions with open-access background-free Lock-in-SIM

Wenjie Liu, Meng Zhang, Wenbin Zhu, Shunyu Xie, Jinfeng Zhang, Shuhao Qian, Zhiyi Liu, Xiu Zheng, Qiuyuan Fang, Wei Yang, Yi Wang, Dazhao Zhu, Jianjie Dong, Xu Liu, Youhua Chen, Cuifang Kuang, Yu-Hui Zhang, Lothar Schermelleh

Correspondence to: wenjie.liu@chem.ox.ac.uk; lothar.schermelleh@bioch.ox.ac.uk; zhangyh@mail.hust.edu.cn; chenyh21012@zju.edu.cn

The file includes:

Supplementary Notes 1 – 3

Supplementary Figures 1 – 36

Supplementary Tables 1-2

Supplementary References

Supplementary Note 1: Principle and workflow of Lock-in-SIM

Lock-in-SIM extracts the in-focused signal from the raw images based on the characteristic that the in-focused signal has a higher modulation contrast than the background. The detailed principle and workflow of Lock-in-SIM are as follows.

Step 1. Determining the illumination pattern parameters

In SIM imaging, the fluorescent sample is excited by sinusoidal illumination patterns. In general, a typical 2D-SIM raw dataset contains nine images corresponding to three pattern orientations and three phases.

In a given pattern orientation, the relationship between the fluorescent sample $O(\mathbf{r})$, the illumination pattern, and the raw image $I_j(\mathbf{r})$ can be expressed as follows:

$$\begin{aligned} I_j(\mathbf{r}) &= \{[1 + mcos(\mathbf{k}_0\mathbf{r} + \varphi_j)] \cdot O(\mathbf{r})\} * H(\mathbf{r}) \\ &= \{O_{out}(\mathbf{r}) + [1 + mcos(\mathbf{k}_0\mathbf{r} + \varphi_j)] \cdot O_{in}(\mathbf{r})\} * H(\mathbf{r}) \end{aligned} \quad (1)$$

where $\mathbf{k}_0$ represents the modulation frequency, φ_j is the jth phase of the illumination pattern (j=1,2,3), m is the modulation depth, $O_{in}(\mathbf{r})$ represents the in-focused signal, $O_{out}(\mathbf{r})$ represents the background, $H(\mathbf{r})$ is the system PSF, and * denotes the convolution operation. In this work, we mainly consider background as unmodulated or poorly modulated[1-3], accounting for substantial contributions from unmodulated components, such as the background originating from light scattering, autofluorescence, and out-of-focus blur from deep sample regions.

In the frequency domain, the raw SIM data in one orientation can be expressed in the following matrix form:

$$\begin{bmatrix} \tilde{I}_1(\mathbf{k}) \\ \tilde{I}_2(\mathbf{k}) \\ \tilde{I}_3(\mathbf{k}) \end{bmatrix} = \begin{bmatrix} 1 & \frac{m}{2}e^{i\varphi_1} & \frac{m}{2}e^{-i\varphi_1} \\ 1 & \frac{m}{2}e^{i\varphi_2} & \frac{m}{2}e^{-i\varphi_2} \\ 1 & \frac{m}{2}e^{i\varphi_3} & \frac{m}{2}e^{-i\varphi_3} \end{bmatrix} \cdot \begin{bmatrix} \tilde{O}(\mathbf{k}) \cdot \tilde{H}(\mathbf{k}) \\ \tilde{O}(\mathbf{k} - \mathbf{k_0}) \cdot \tilde{H}(\mathbf{k}) \\ \tilde{O}(\mathbf{k} + \mathbf{k_0}) \cdot \tilde{H}(\mathbf{k}) \end{bmatrix} \quad (2)$$

where ~ represents the Fourier transform operation.

To estimate the pattern parameters $\mathbf{k}_0$ and φ_j, we can derive the preliminary separated frequency components $\tilde{S}_i(\mathbf{k})$ by introducing a demodulation matrix M_d:

$$\begin{bmatrix} \tilde{S}_1(\mathbf{k}) \\ \tilde{S}_2(\mathbf{k}) \\ \tilde{S}_3(\mathbf{k}) \end{bmatrix} = {M_d}^{-1} \cdot \begin{bmatrix} \tilde{I}_1(\mathbf{k}) \\ \tilde{I}_2(\mathbf{k}) \\ \tilde{I}_3(\mathbf{k}) \end{bmatrix}$$

$$= \begin{bmatrix} a_1 & \frac{m}{2}e^{i\alpha_1} & \frac{m}{2}e^{-i\alpha_1} \\ a_2 & \frac{m}{2}e^{i\alpha_2} & \frac{m}{2}e^{-i\alpha_2} \\ a_3 & \frac{m}{2}e^{i\alpha_3} & \frac{m}{2}e^{-\alpha_3} \end{bmatrix}^{-1} \cdot \begin{bmatrix} 1 & \frac{m}{2}e^{i\varphi_1} & \frac{m}{2}e^{-i\varphi_1} \\ 1 & \frac{m}{2}e^{i\varphi_2} & \frac{m}{2}e^{-i\varphi_2} \\ 1 & \frac{m}{2}e^{i\varphi_3} & \frac{m}{2}e^{-i\varphi_3} \end{bmatrix} \cdot \begin{bmatrix} \tilde{O}(\mathbf{k}) \cdot \tilde{H}(\mathbf{k}) \\ \tilde{O}(\mathbf{k} - \mathbf{k_0}) \cdot \tilde{H}(\mathbf{k}) \\ \tilde{O}(\mathbf{k} + \mathbf{k_0}) \cdot \tilde{H}(\mathbf{k}) \end{bmatrix}$$

$$= \begin{bmatrix} b_1 & b_2 & b_3 \\ c_1e^{i\beta_1} & c_2e^{i\beta_2} & c_3e^{i\beta_3} \\ c_1e^{-i\beta_1} & c_2e^{-i\beta_2} & c_3e^{-i\beta_3} \end{bmatrix} \cdot \begin{bmatrix} 1 & \frac{m}{2}e^{i\varphi_1} & \frac{m}{2}e^{-i\varphi_1} \\ 1 & \frac{m}{2}e^{i\varphi_2} & \frac{m}{2}e^{-i\varphi_2} \\ 1 & \frac{m}{2}e^{i\varphi_3} & \frac{m}{2}e^{-i\varphi_3} \end{bmatrix} \cdot \begin{bmatrix} \tilde{O}(\mathbf{k}) \cdot \tilde{H}(\mathbf{k}) \\ \tilde{O}(\mathbf{k}-\mathbf{k_0}) \cdot \tilde{H}(\mathbf{k}) \\ \tilde{O}(\mathbf{k}+\mathbf{k_0}) \cdot \tilde{H}(\mathbf{k}) \end{bmatrix}$$

$$= \begin{bmatrix} d_1 & \gamma_1 & conj(\gamma_1) \\ \gamma_2 & d_2e^{i\emptyset_1} & d_3e^{i\emptyset_2} \\ conj(\gamma_2) & d_3e^{-i\emptyset_2} & d_2e^{-i\emptyset_1} \end{bmatrix} \cdot \begin{bmatrix} \tilde{O}(\mathbf{k}) \cdot \tilde{H}(\mathbf{k}) \\ \tilde{O}(\mathbf{k}-\mathbf{k_0}) \cdot \tilde{H}(\mathbf{k}) \\ \tilde{O}(\mathbf{k}+\mathbf{k_0}) \cdot \tilde{H}(\mathbf{k}) \end{bmatrix} \quad (3)$$

where $conj()$ represents the conjugation operation, γ_1 and γ_2 are complex numbers, and a_j, b_j, c_j, d_j, α_j, β_j (j=1,2,3), $\emptyset_1$, $\emptyset_2$ are real numbers resulting from matrix introduction and multiplication.

To estimate $\mathbf{k}_0$, (a_1, a_2, a_3) and $(\alpha_1, \alpha_2, \alpha_3)$ were first ideally chosen as (1,1,1) and (0, 2π/3, 4π/3), respectively. Then, $\mathbf{k}_0$ can be calculated by performing phase-only correlation and identifying the peak coordinates of the following correlation result:

$$\frac{\widetilde{WF}(\mathbf{k})}{|\widetilde{WF}(\mathbf{k})|+\delta} \otimes \frac{\tilde{S}_2(\mathbf{k})}{|\tilde{S}_2(\mathbf{k})|+\delta} = \int conj\left[\frac{\widetilde{WF}(\mathbf{k})}{|\widetilde{WF}(\mathbf{k})|+\delta}\right] \cdot \frac{\tilde{S}_2(\mathbf{k}+\mathbf{k_0})}{|\tilde{S}_2(\mathbf{k}+\mathbf{k_0})|+\delta} d\mathbf{k} \quad (4)$$

where $\otimes$ represents the correlation operation, $\widetilde{WF}(\mathbf{k})$ is widefield frequency, and δ is a small positive constant.

Thus, the phase of the above correlation function, which is equivalent to $\emptyset_1$ in Eq. (3), can be obtained by calculating its argument:

$$\arg\left[\widetilde{WF}(\mathbf{k})\otimes\tilde{S}_2(\mathbf{k})\right] = \arg\left\{\int conj\left[\widetilde{WF}(\boldsymbol{k})\right] \cdot \tilde{S}_2(\boldsymbol{k}+\boldsymbol{k_0}) d\boldsymbol{k}\right\} \quad (5)$$

Then to estimate φ_j, we revisit Eq. (3) and have:

$$d_2e^{i\emptyset_1} = \frac{m}{2}\left[c_1e^{i(\varphi_1+\beta_1)} + c_2e^{i(\varphi_2+\beta_2)} + c_3e^{i(\varphi_3+\beta_3)}\right] \quad (6)$$

Eq. (6) can be transformed as:

$$\sum_{j=1}^{3} c_j sin(\emptyset_1 - \beta_j - \varphi_j) = 0 \quad (7)$$

By further introducing multiple demodulation matrices M_d to obtain independent trigonometric equations, φ_j can finally be solved. This matrix-based parameter estimation approach is fast and robust, building upon our previous work[4], which readers can refer to for more details. Other existing methods could also be utilized to estimate the illumination parameters. By repeating the above processes for all three angles, the parameters of all illumination patterns could be determined.

Step 2. Lock-in demodulation

The in-focused signal and background can also be expressed as the modulation-dependent (AC) component I_{ac} (in-focused signal) and modulation-independent (DC) component I_{dc} (background), respectively. Thus, the raw image can be represented as the sum of the DC and AC components:

$$I_j(\mathbf{r}) = [O_{out}(\mathbf{r}) + (1-m) \cdot O_{in}(\mathbf{r})] * H(\mathbf{r}) + \left[m + mcos(\mathbf{k_0 r} + \varphi_j)\right] \cdot O_{in}(\mathbf{r}) * H(\mathbf{r})$$

$$= I_{dc}(\mathbf{r}) + I_{j_{ac}}(\mathbf{r}) \tag{8}$$

To calculate the DC component I_{dc}, it can be rewritten as follows:

$$\begin{aligned} I_{dc}(\mathbf{r}) &= [O_{out}(\mathbf{r}) + (1-m)\cdot O_{in}(\mathbf{r})] * H(\mathbf{r}) \\ &= [O_{out}(\mathbf{r}) + O_{in}(\mathbf{r})] * H(\mathbf{r}) - mO_{in}(\mathbf{r}) * H(\mathbf{r}) \\ &= I_{dc1}(\mathbf{r}) - I_{dc2}(\mathbf{r}) \end{aligned} \tag{9}$$

The new two DC components $I_{dc1}(\mathbf{r})$ and $I_{dc2}(\mathbf{r})$ can then be obtained directly from raw images $I_j(\mathbf{r})$ at three phases:

$$I_{dc1}(\mathbf{r}) = [O_{out}(\mathbf{r}) + O_{in}(\mathbf{r})] * H(\mathbf{r}) = \frac{1}{3}\sum_{j=1}^{3} I_j(\mathbf{r}) \tag{10}$$

$$I_{dc2}(\mathbf{r}) = mO_{in}(\mathbf{r}) * H(\mathbf{r}) = \sqrt{\frac{2}{9}\sum_{j_1,j_2}\left[I_{j_1}(\mathbf{r}) - I_{j_2}(\mathbf{r})\right]^2} \tag{11}$$

where j_1, j_2 belongs to [1,2,3].

As a result, the background-filtered raw image after Lock-in demodulation is derived as follows:

$$\begin{aligned} I_{j_{lcok-in}}(\mathbf{r}) &= \{[1 + m\cos(\mathbf{k}_0\mathbf{r} + \varphi_j)]\cdot O_{in}(\mathbf{r})\} * H(\mathbf{r}) \\ &= I_j(\mathbf{r}) - I_{dc1}(\mathbf{r}) + \frac{1}{m} I_{dc2}(\mathbf{r}) \\ &\approx I_j(\mathbf{r}) - \varepsilon\frac{1}{3}\sum_{j=1}^{3} I_j(\mathbf{r}) + \sqrt{\frac{2}{9}\sum_{j_1,j_2}\left[I_{j_1}(\mathbf{r}) - I_{j_2}(\mathbf{r})\right]^2} \end{aligned} \tag{12}$$

where ε is a balanced weight and ranges from 0 to 1. The empirical value of ε is 0.8. The modulation depth *m* is omitted at this step to simplify the processing, without compromising the reconstruction quality.

Note that although Eq. (11) resembles standard OS-SIM[5], OS-SIM does not preserve the modulation pattern and therefore cannot achieve super-resolution reconstruction. In contrast, Lock-in-SIM filtering preserves both the modulation pattern and in-focused signal effectively.

Step 3. Super-resolution reconstruction

With the Lock-in filtered raw data, Eq. (2) can be updated as:

$$\begin{bmatrix} \tilde{I}_{1_{lock-in}}(\mathbf{k}) \\ \tilde{I}_{2_{lock-in}}(\mathbf{k}) \\ \tilde{I}_{3_{lock-in}}(\mathbf{k}) \end{bmatrix} = \begin{bmatrix} 1 & \frac{m}{2}e^{i\varphi_1} & \frac{m}{2}e^{-i\varphi_1} \\ 1 & \frac{m}{2}e^{i\varphi_2} & \frac{m}{2}e^{-i\varphi_2} \\ 1 & \frac{m}{2}e^{i\varphi_3} & \frac{m}{2}e^{-i\varphi_3} \end{bmatrix} \cdot \begin{bmatrix} \widetilde{O_{in}}(\mathbf{k})\cdot\widetilde{H}(\mathbf{k}) \\ \widetilde{O_{in}}(\mathbf{k}-\mathbf{k_0})\cdot\widetilde{H}(\mathbf{k}) \\ \widetilde{O_{in}}(\mathbf{k}+\mathbf{k_0})\cdot\widetilde{H}(\mathbf{k}) \end{bmatrix} \tag{13}$$

Since the illumination parameters were determined in the first step, the high-frequency components and low-frequency components could be calculated and separated as follows:

$$\begin{bmatrix} \widetilde{O_{in}}(\mathbf{k})\cdot\widetilde{H}(\mathbf{k}) \\ \widetilde{O_{in}}(\mathbf{k}-\mathbf{k_0})\cdot\widetilde{H}(\mathbf{k}) \\ \widetilde{O_{in}}(\mathbf{k}+\mathbf{k_0})\cdot\widetilde{H}(\mathbf{k}) \end{bmatrix} = \begin{bmatrix} 1 & \frac{m}{2}e^{i\varphi_1} & \frac{m}{2}e^{-i\varphi_1} \\ 1 & \frac{m}{2}e^{i\varphi_2} & \frac{m}{2}e^{-i\varphi_2} \\ 1 & \frac{m}{2}e^{i\varphi_3} & \frac{m}{2}e^{-i\varphi_3} \end{bmatrix}^{-1} \begin{bmatrix} \tilde{I}_{1_{lock-in}}(\mathbf{k}) \\ \tilde{I}_{2_{lock-in}}(\mathbf{k}) \\ \tilde{I}_{3_{lock-in}}(\mathbf{k}) \end{bmatrix} \tag{14}$$

By shifting the high-frequency components to corresponding positions according to $\mathbf{k}_0$ and combining the shifted spectra through common apodized Wiener filtering, the frequency distribution of the super-resolution Lock-in-SIM result $SR_{lock-in}$ at one orientation can be

obtained:

$$\widetilde{SR}_{lock-in} = \frac{\sum_{m=0,1,-1} \widetilde{O_{in}}(\mathbf{k}+\mathbf{mk_0}) \cdot \widetilde{H}(\mathbf{k}) \cdot conj[\widetilde{H}(\mathbf{k}+\mathbf{mk_0})]}{\sum_{m=0,1,-1} \left|\widetilde{H}(\mathbf{k}+\mathbf{mk_0})\right|^2 + \omega^2} \cdot \tilde{A}(\mathbf{k}) \tag{15}$$

where $\tilde{A}(\mathbf{k})$ is an apodization function to compensate for possible ringing artifacts. By combining results from all three orientations and performing inverse Fourier transform, $SR_{lock-in}$ is derived. Finally, post-deconvolution and filtering can be applied to further improve image quality.

Supplementary Note 2: Lock-in-SIM demodulation based on cross-correlation analysis

In conventional lock-in detection methods, a cross-correlation operation is commonly used to distinguish modulated signals from background noise. The highly correlated reference signal could be obtained internally by tracking the fluorescence fluctuation of a small representative region or externally by recording the fluorescence fluctuation of a standard sample[6].

When applying cross-correlation analysis to our Lock-in-SIM, the reference waveform could also be recorded externally by, for example, replacing the sample with a reflective mirror. Nevertheless, the external recording approach is troublesome and may not be accurate because the illumination pattern is affected by the characteristics of the samples and systems. In contrast, the reference waveform should be generated internally since the parameters of the illumination pattern have been determined in Step 1 above (Supplementary Note 1).

With the determined pattern $P_j(\mathbf{r})$ and raw image $I_j(\mathbf{r})$, we can calculate the cross-correlation coefficient:

$$\rho(\mathbf{r}) = \sum_{j=1}^{3} \frac{[I_j(\mathbf{r})-\mu_I][P_j(\mathbf{r})-\mu_P]}{\sigma_I\sigma_P} \quad (16)$$

where μ_I and σ_I are the mean value and standard deviation value of the pixel fluorescence intensity of $I_j(\mathbf{r})$, respectively. μ_P and σ_P are the mean value and standard deviation value of the pixel fluorescence intensity of $P_j(\mathbf{r})$, respectively. The cross-correlation coefficient $\rho(\mathbf{r})$ represents the correlation degree between the sample structure and the sinusoidal illumination pattern, with a larger coefficient denoting a greater proportion of the in-focused signal at a certain pixel.

After obtaining the correlation coefficient map, the background-filtered raw image with Lock-in demodulation can be obtained by multiplying the correlation coefficients and raw images pixel by pixel:

$$I_{j_{lcok-in}}(\mathbf{r}) = \rho(\mathbf{r}) \cdot I_j(\mathbf{r}) = \sum_{j=1}^{3} \frac{[I_j(\mathbf{r})-\mu_I][P_j(\mathbf{r})-\mu_P]}{\sigma_I\sigma_P} \cdot I_j(\mathbf{r}) \quad (17)$$

The above calculation procedure could be directly used to replace Step 2, as stated in Supplementary Note 1, to achieve conventional correlation-based Lock-in-SIM reconstruction (Supplementary Fig. 1). However, we found that cross-correlation analysis was not suitable for selectively amplifying signals from raw SIM data in our test (Supplementary Fig. 1) as it typically requires hundreds of raw images for precise correlation analysis[6]. Besides, other unmodulated background sources, such as the background from uneven light scattering, autofluorescence, and deep sample regions, might also affect this correlation relationship. Therefore, we developed a direct DC and AC separation method, as explained in Supplementary Note 1, to achieve high-quality and fast Lock-in-SIM demodulation.

Supplementary Note 3: Comparison with other background-filtering SIM algorithms

Several algorithms, such as LROS-SIM[7] (also known as integrated-SIM[8]), Max-Kz-SIM[7], WLR-SIM[7], BF-SIM[9], direct-SIM[10], JSFR-SIM[11], and Flex-SIM[12], have been developed to filter background in super-resolution SIM imaging. However, they still face challenges in balancing background suppression, structural integrity, ultrastructure preservation, and image fidelity.

The underlying background-filtering principle of LROS-SIM, Max-Kz-SIM, and WLR-SIM is the same, that is, selecting the high-frequency components (±1-order) in the frequency domain for band combination and extension. While in our Lock-in-SIM, the background and signal are separated directly in the spatial domain, followed by the standard SIM reconstruction in the frequency domain. The crucial limitation of these three methods is that extracting two high-frequency components for combination in the frequency domain will result in artifacts in the low-frequency band, thus restricting their effective applications to half the frequency extension[7]. Consequently, when applying these methods to standard high-frequency extension SIM data, artifacts are prone to be introduced (Extended Fig. 2 and Supplementary Fig. 7). In contrast, our Lock-in-SIM does not have this trade-off and achieves superior background-suppressed super-resolution reconstruction quality across various challenging experimental conditions.

BF-SIM treats the PSF as a 3D distribution rather than the commonly used 2D model (as in Lock-in-SIM) in 2D-SIM imaging. By generating a simulated 3D PSF based on system parameters including the immersion refractive index, wavelength, numerical aperture, and pixel size, and subsequently subtracting the out-of-focus PSF contribution, BF-SIM achieves effective background suppression. However, accurately modeling an actual 3D PSF in simulation to align with complicated experimental conditions is challenging. Any mismatch, for example due to optical aberrations, may compromise the reconstruction quality.

Direct-SIM proposed a spatial super-resolution reconstruction approach instead of the standard frequency SIM reconstruction preceding the post HiFi filtering stage. Although the spatial reconstruction equation used in Direct-SIM appears similar in form to the OS-SIM equation[5] and our equation (Supplementary Eq. (11)), the underlying principles differ significantly. Direct-SIM employs multiple approximations in deriving its equations, which may compromise reconstruction quality under certain conditions (Extended Fig. 3 and Supplementary Figs. 7,8,13). Besides, Direct-SIM relied on multiple weight factors to balance the reconstruction quality, imposing a high burden for post-parameter finetuning.

JSFR-SIM also utilized spatial super-resolution reconstruction to improve the processing speed significantly. However, similar to Direct-SIM, it is susceptible to artifacts. Flex-SIM implemented several modifications to refine conventional SIM reconstruction. Nonetheless, as demonstrated in this study, striking a good balance between background suppression and image fidelity remains difficult.

Supplementary Figures

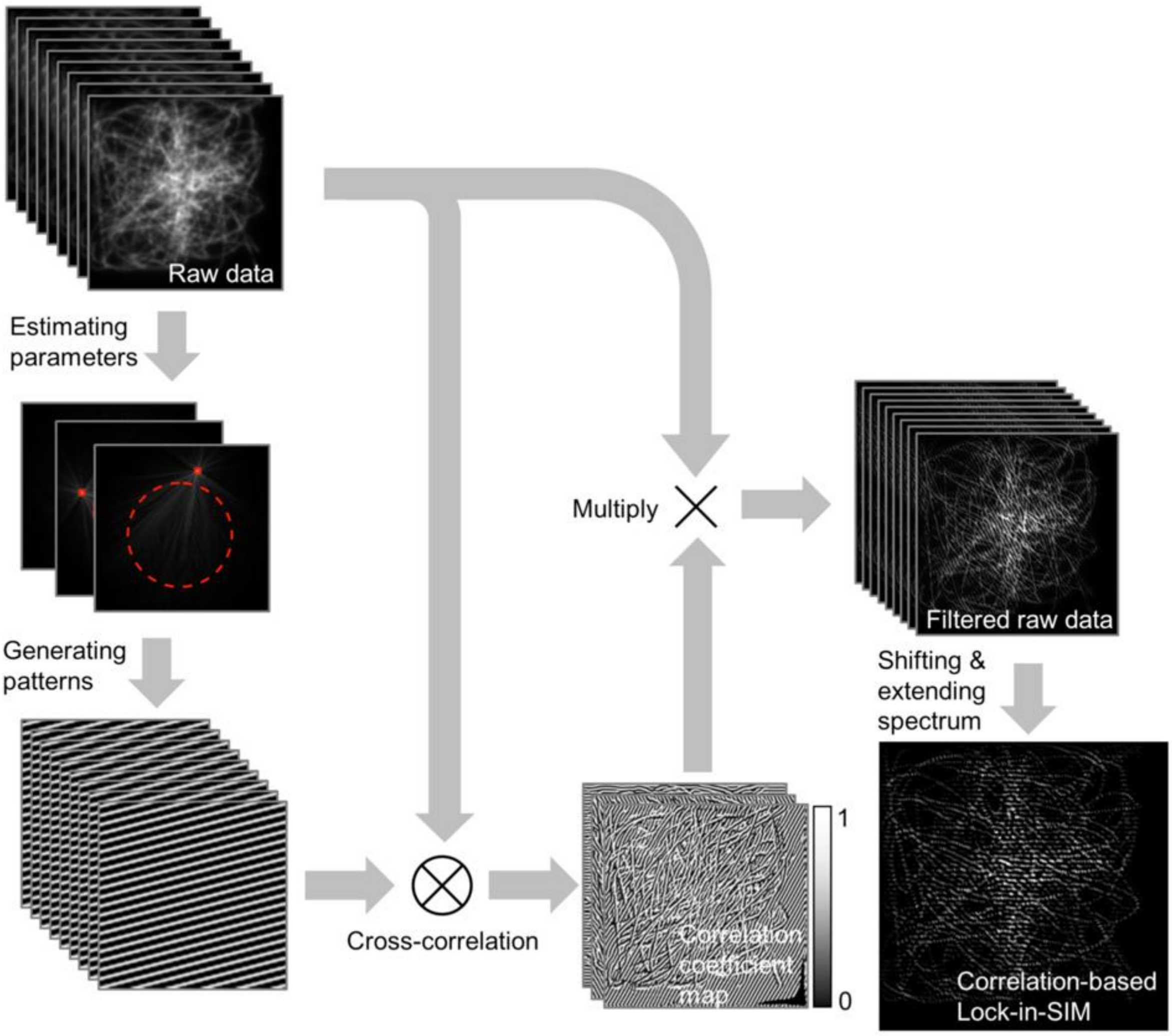

Supplementary Fig. 1. Reconstruction flowchart of correlation-based Lock-in-SIM. The reconstruction input consists of conventional 9 raw SIM images acquired at different angles and phases. The illumination parameters are then estimated to generate the corresponding patterns, which are cross-correlated with the raw data to produce correlation coefficient maps. Thus, the background can be removed by multiplying the raw images with correlation coefficients pixel by pixel. Finally, the SIM result is obtained after shifting and extending the frequency spectrum of the filtered raw data with the estimated parameters in the first step. Note that the reconstruction flowchart can also be directly applied to 3D-SIM data.

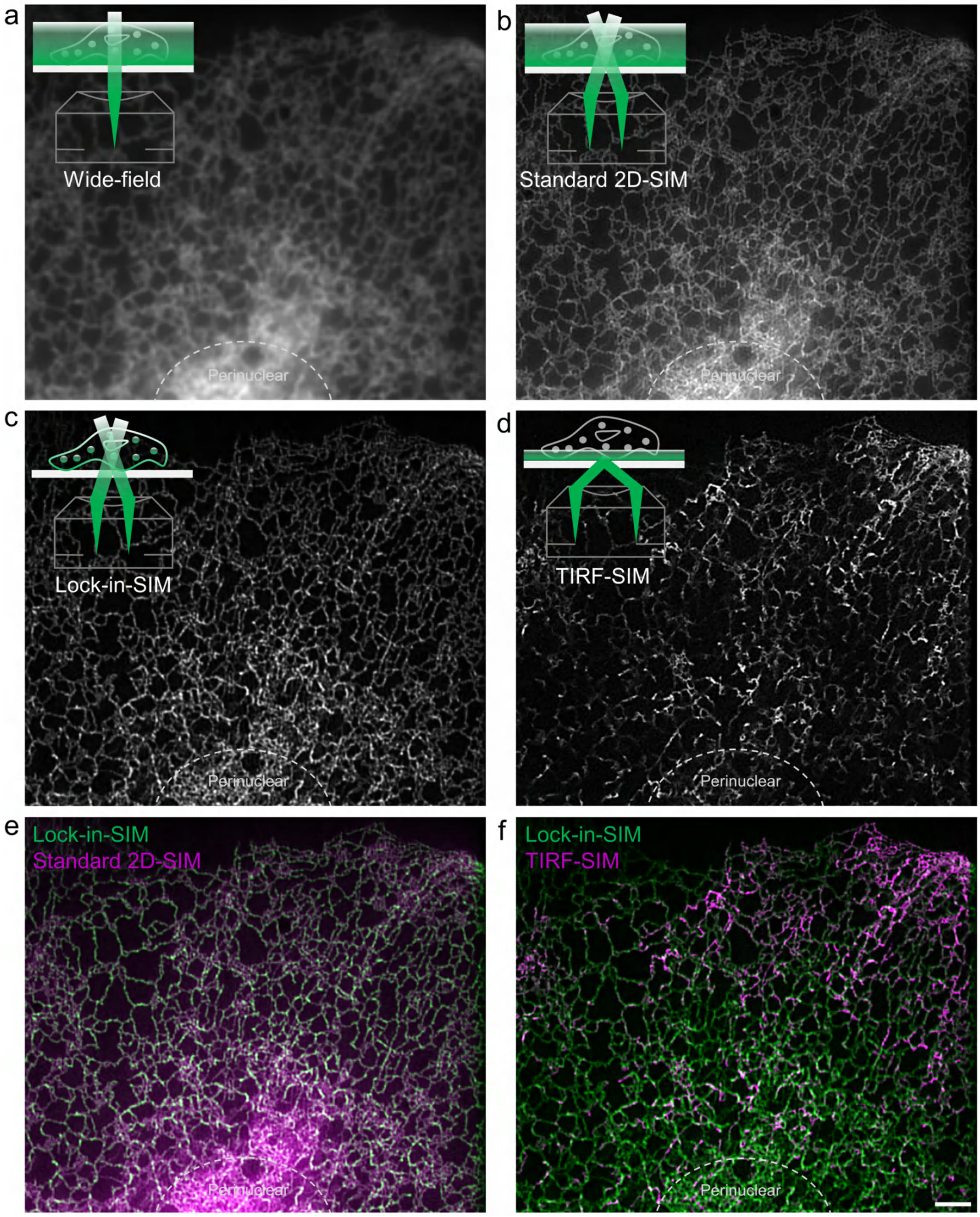

Supplementary Fig. 2. Reconstruction comparison of the thick cell sample using different 2D-SIM methods. a-d, Experimental ER (labeled with Sec61β-EGFP) results from widefield microscopy (**a**), standard 2D-SIM (**b**), Lock-in-SIM (**c**), and TIRF-SIM (**d**). The upper-left diagram shows the corresponding illumination scheme. **e,f**, Superposition of Lock-in-SIM image (green) with standard 2D-SIM image (magenta) (**e**) and Lock-in-SIM image (green) with TIRF-SIM image (magenta) (**f**). The dotted curves denote the thicker and denser perinuclear region. Scale bars, 4 μm (**a-f**).

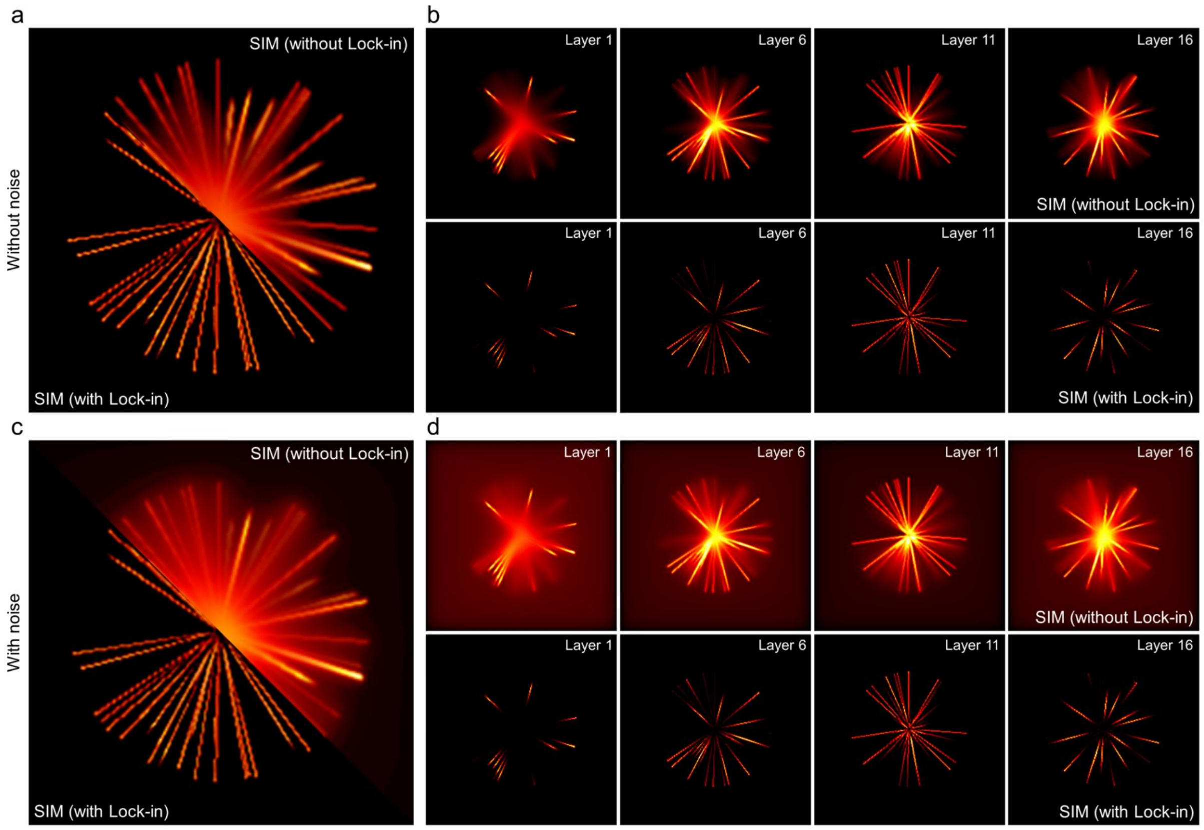

Supplementary Fig. 3. Background-free, optical-sectioning, and noise-robust reconstruction of Lock-in-SIM using a simulated 3D volumetric filament sample. a, Maximum intensity projection of layered super-resolution images reconstructed without (upper right) and with (bottom left) Lock-in filtering without the influence of noise. **b**, Reconstruction results for different layer depths without the influence of noise. **c**, Maximum intensity projection of layered super-resolution images reconstructed without (upper right) and with (bottom left) Lock-in filtering under the influence of noise. **d**, Reconstruction results for different layer depths under the influence of noise.

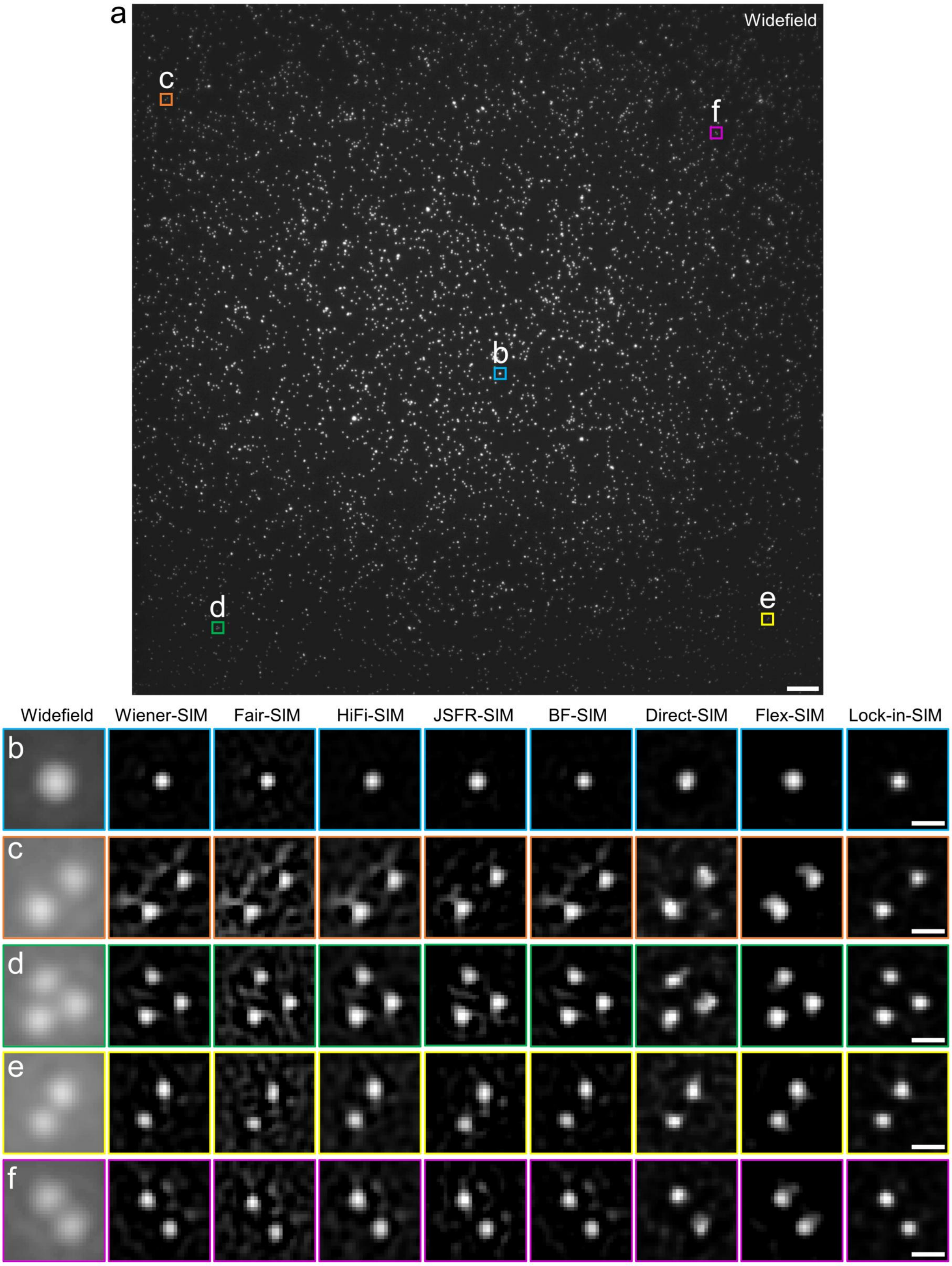

Supplementary Fig. 4. Reconstruction comparison of large-field-of-view 100-nm fluorescent beads using different 2D-SIM algorithms. **a**, Large-field-of-view widefield image of beads (108 μm*108 μm), with peripheral regions typically exhibiting possible higher illumination distortion. **b-f**, Magnified images of the corresponding color boxed regions in **a** reconstructed using different 2D-SIM methods, showing the robust reconstruction capability of Lock-in-SIM. Scale bars, 5 μm (**a**) and 0.3 μm (**b-f**).

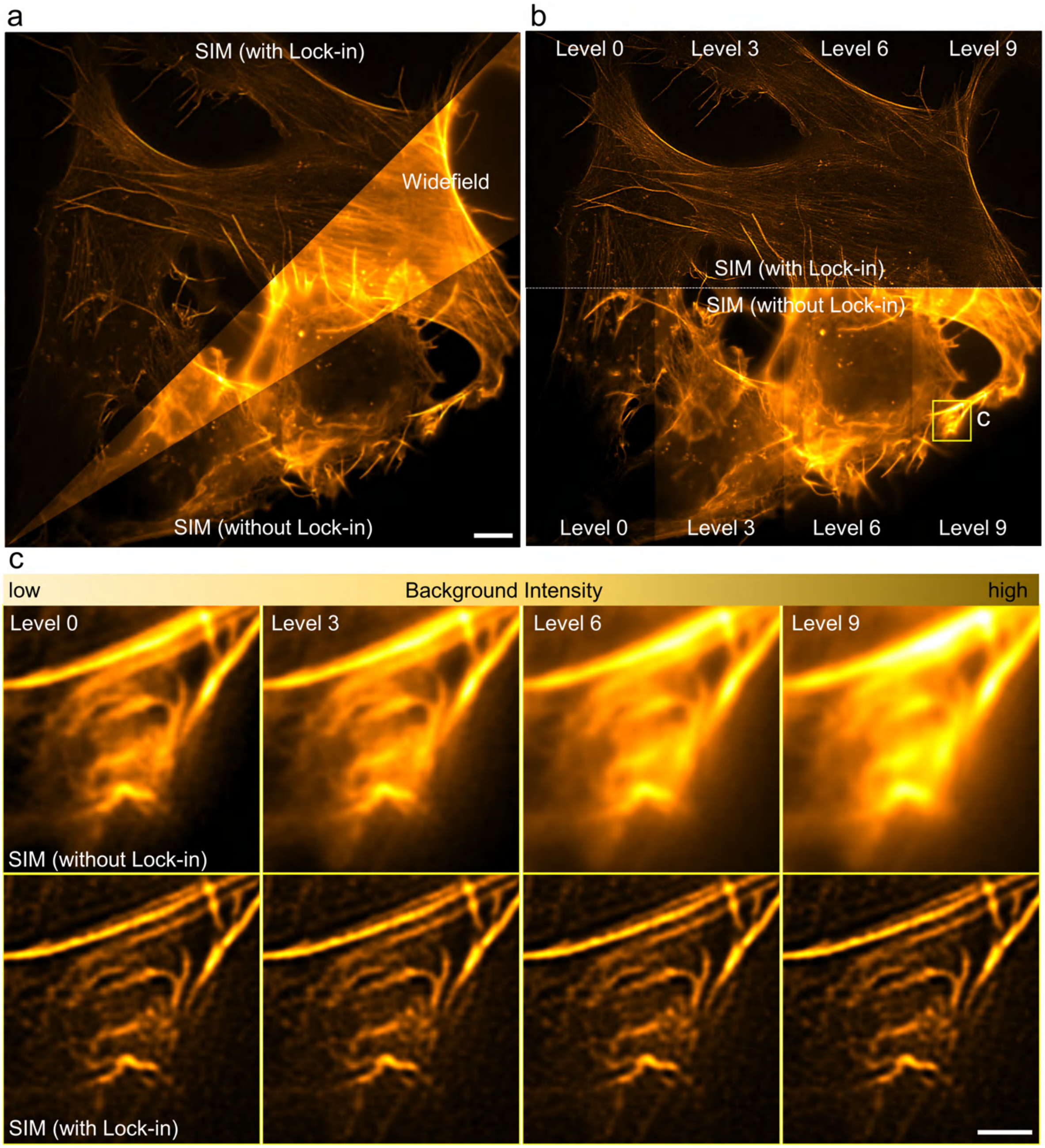

Supplementary Fig. 5. Background-free and -robust reconstruction of Lock-in-SIM using a synthetic actin sample. a, Widefield image and super-resolution images reconstructed without (bottom right) and with (upper left) Lock-in filtering. **b**, Super-resolution images reconstructed without (bottom) and with (upper) Lock-in filtering under different background levels. Level 0 denotes no additional background. **c**, Magnified images of the yellow boxed region in **b**. Scale bars, 5 μm (**a**,**b**) and 1 μm (**c**).

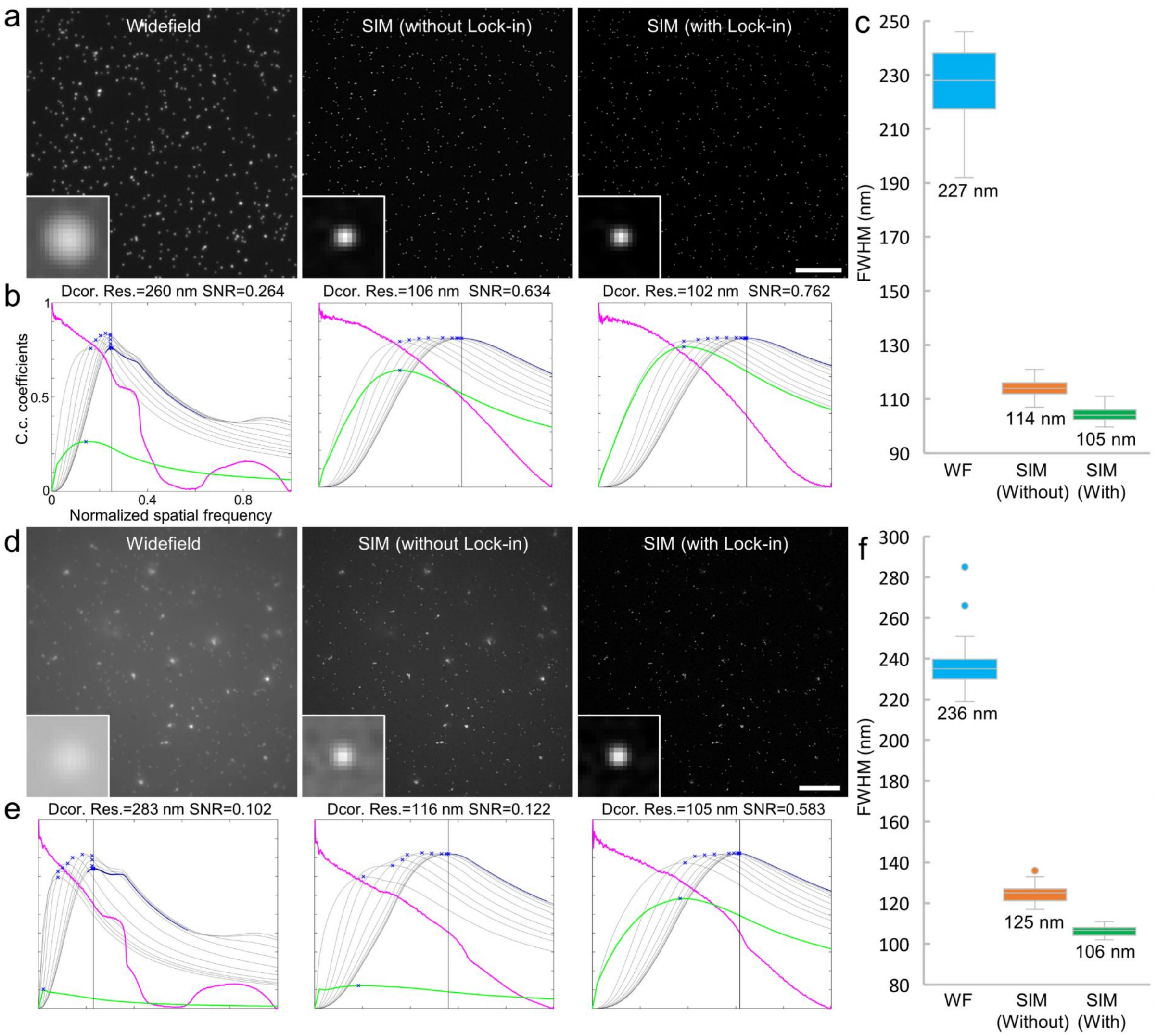

Supplementary Fig. 6. Background-free and -robust reconstruction of Lock-in-SIM using 100-nm fluorescent bead sample. a,d, Widefield image and super-resolution SIM images reconstructed without and with Lock-in filtering of low-background (**a**) and high-background (**d**) raw bead data. Bottom-left insets are the corresponding representative bead PSFs. **b,e**, Corresponding resolution and SNR assessment results outputted from decorrelation analysis of low-background (**b**) and high-background (**e**) bead data. The x- and y- coordinates of all plots are the same. C.c., cross correlation; Dcor., decorrelation; Res., resolution; SNR, signal-to-noise ratio. **c,f**, The corresponding resolution assessment results based on statistical full width at half maximum (FWHM) measurements of low-background (**c,** *n*=48 per group) and high-background (**f,** *n*=43 per group) bead data. The middle line in the box corresponds to the mean value. The upper and lower boundary lines correspond to the 15th and 75th percentiles, respectively. The whiskers represent the min and max values. The value shown below each boxplot represents the average FWHM for the group. The comparison highlights that Lock-in-SIM stably provides superior background and noise suppression and effective resolution. Scale bars, 5 μm (**a,d**).

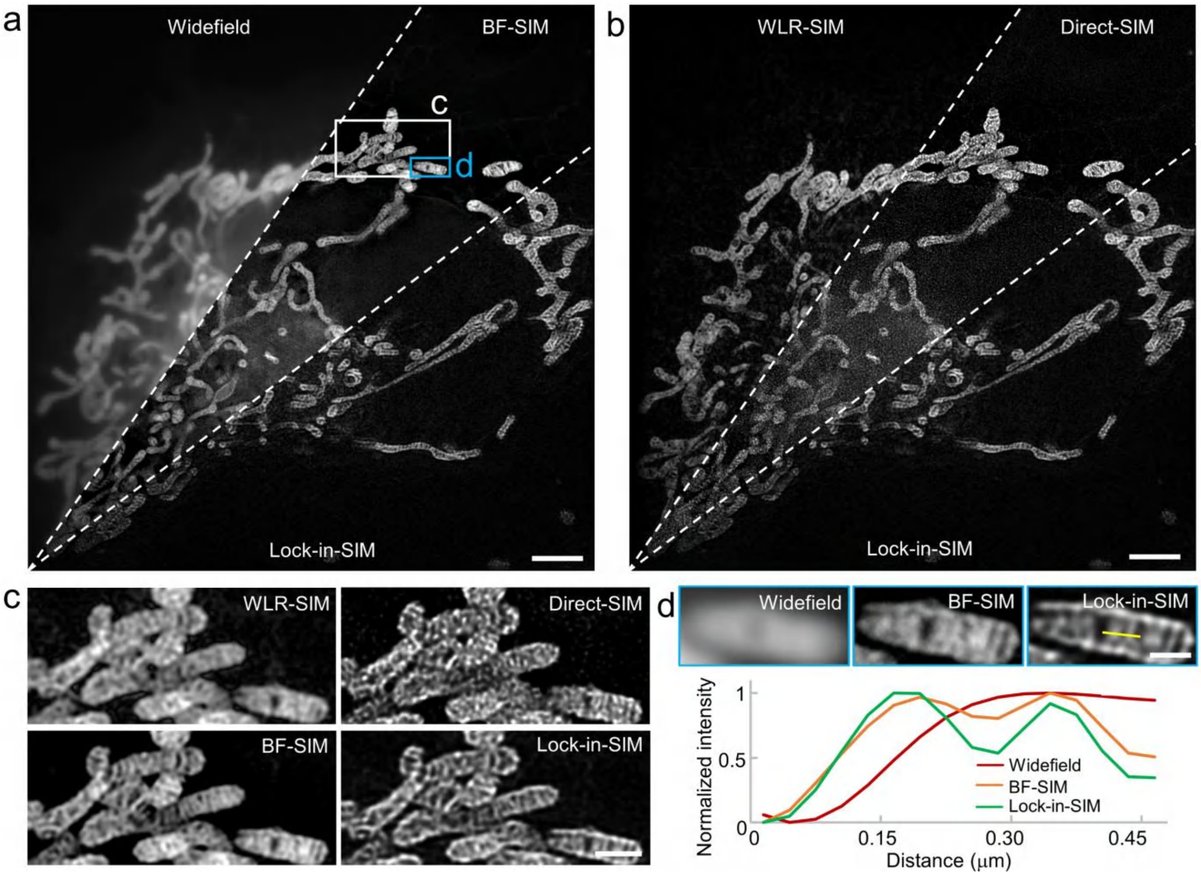

Supplementary Fig. 7. Reconstruction comparison of open-access mitochondria inner membrane data[9] using different 2D-SIM algorithms. **a**, Widefield image (top left) and super-resolution images reconstructed using BF-SIM (middle) and Lock-in-SIM (bottom right). **b**, Super-resolution images reconstructed using WLR-SIM (top left), Direct-SIM (middle) and Lock-in-SIM (bottom right). **c**, Magnified super-resolution images reconstructed using different methods of the white boxed region in **a**. **d**, Top: Magnified widefield, BF-SIM, and Lock-in-SIM images of the blue boxed region in **a**. Bottom: Normalized intensity profiles along the yellow lines. This comparison highlights that Lock-in-SIM provides superior background suppression, membrane definition, and image fidelity. Scale bars, 3 μm (**a,b**), 1 μm (**c**) and 0.5 μm (**d**).

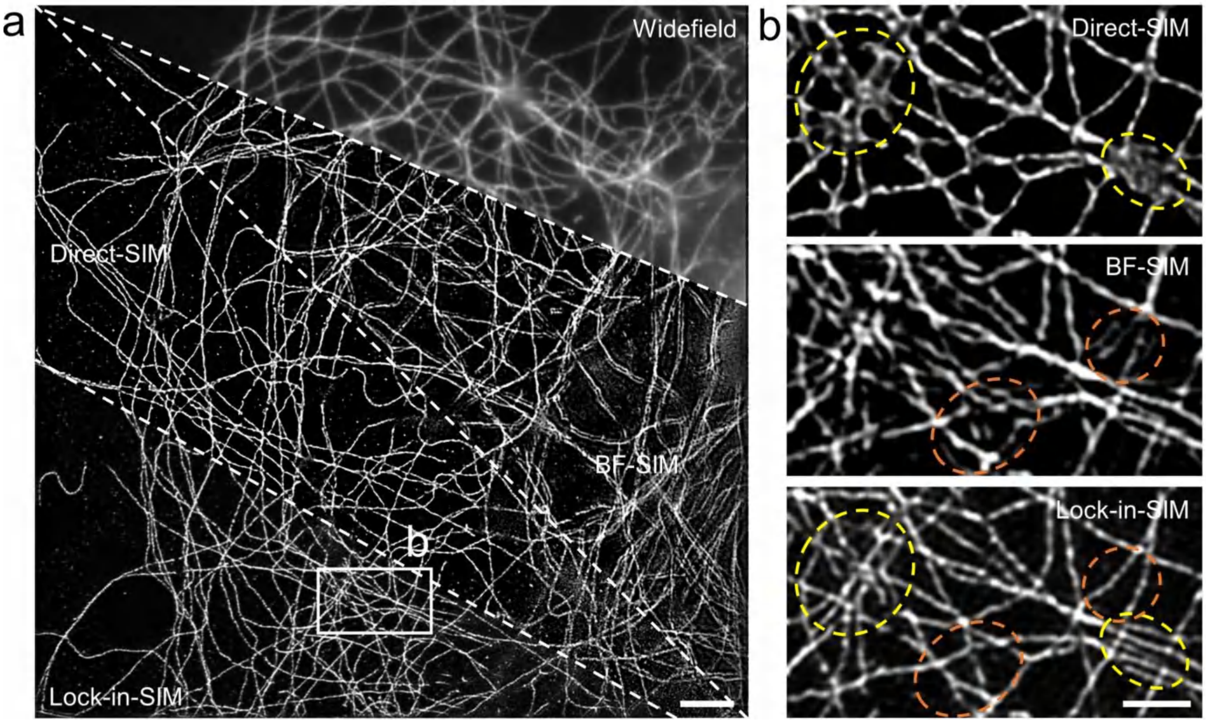

Supplementary Fig. 8. Reconstruction comparison of open-access microtubule data[10] using different 2D-SIM algorithms. a, Widefield image (top right) and corresponding super-resolution images reconstructed using BF-SIM (middle right), Direct-SIM (middle left), and Lock-in-SIM (bottom left). **b**, Magnified super-resolution images reconstructed using different methods of the white boxed regions in **a**. Yellow and orange circles indicate the representative reconstructed results of local ultrastructural regions. This comparison highlights that Lock-in-SIM simultaneously provides superior background suppression, ultrastructure resolution, and image fidelity. Scale bars, 3 μm (**a,b**) and 1 μm (**c**).

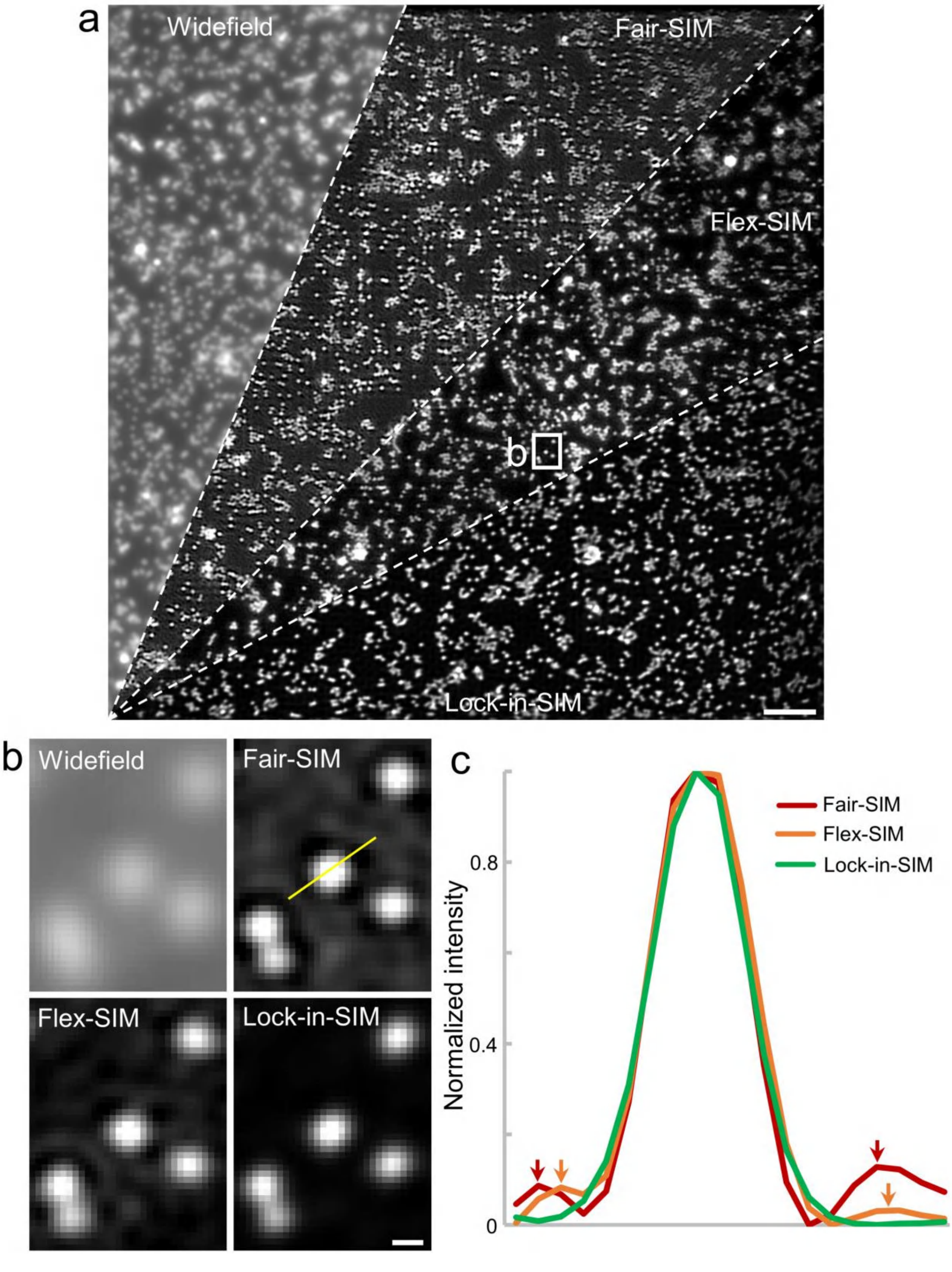

Supplementary Fig. 9. Reconstruction comparison of open-access fluorescent bead data[12,17] using different 2D-SIM algorithms. a, Widefield image (top left) and corresponding super-resolution images reconstructed using Fair-SIM (middle left), Flex-SIM (middle right), and Lock-in-SIM (bottom right). **b**, Magnified widefield and super-resolution images reconstructed using different methods of the white boxed regions in **a**, showing the superior background suppression and image fidelity of Lock-in-SIM. **c**, Normalized intensity profiles along the yellow lines in **b**. Arrows indicate the background artifacts in Fair-SIM and Flex-SIM images. Scale bars, 3 μm (**a**) and 0.2 μm (**b**).

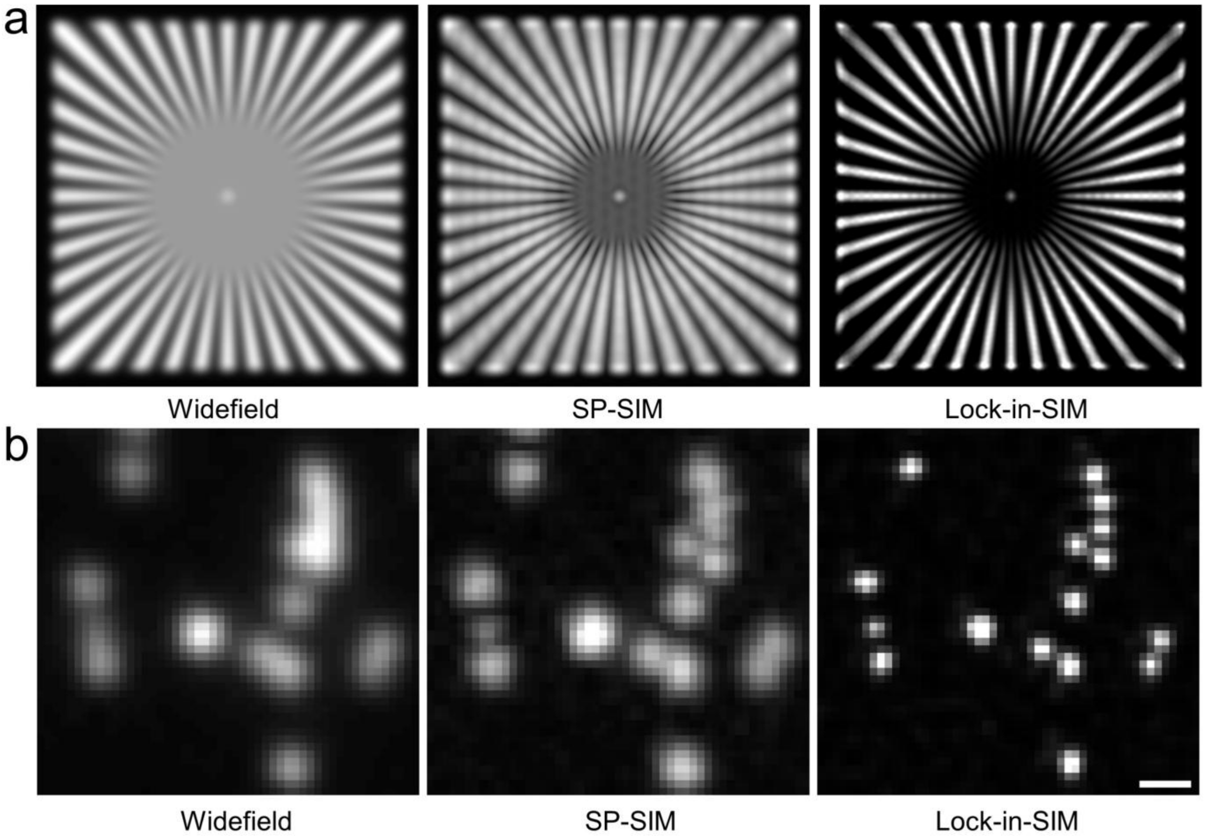

Supplementary Fig. 10. Reconstruction comparison of open-access simulated and experimental data[19] using different 2D-SIM algorithms. **a**, Widefield image (left) of simulated data and corresponding super-resolution images reconstructed using SP-SIM (middle) and Lock-in-SIM (right). **b**, Widefield image (left) of fluorescent beads and corresponding super-resolution images reconstructed using SP-SIM (middle) and Lock-in-SIM (right). The comparison highlights that Lock-in-SIM provides superior background suppression and ultrastructure resolution. Scale bar, 0.3 μm (**b**).

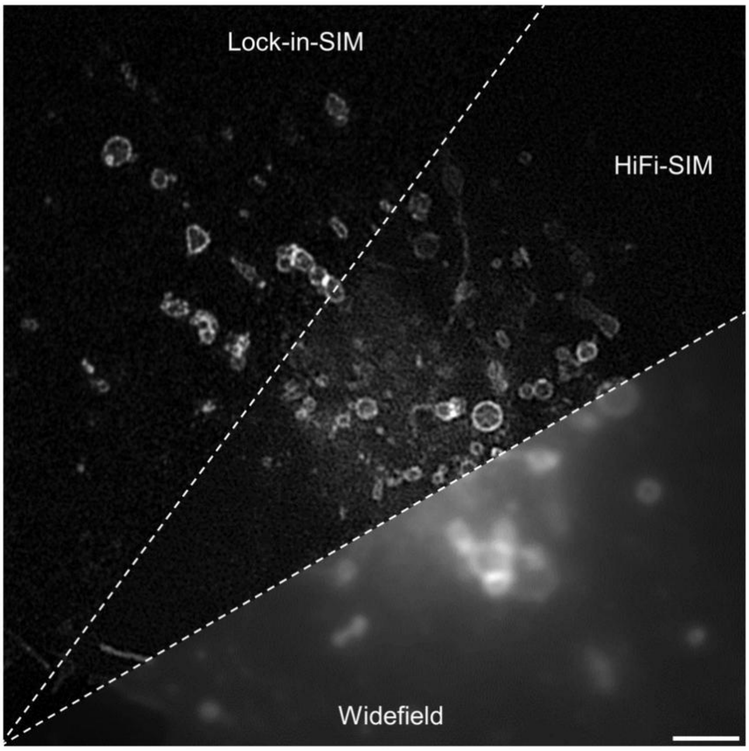

Supplementary Fig. 11. Reconstruction comparison of open-access lysosome data[1] using different 2D-SIM algorithms. Widefield image (bottom) and super-resolution images reconstructed using HiFi-SIM (middle), and Lock-in-SIM (top), showing the superior background suppression of Lock-in-SIM. Scale bar, 2 μm.

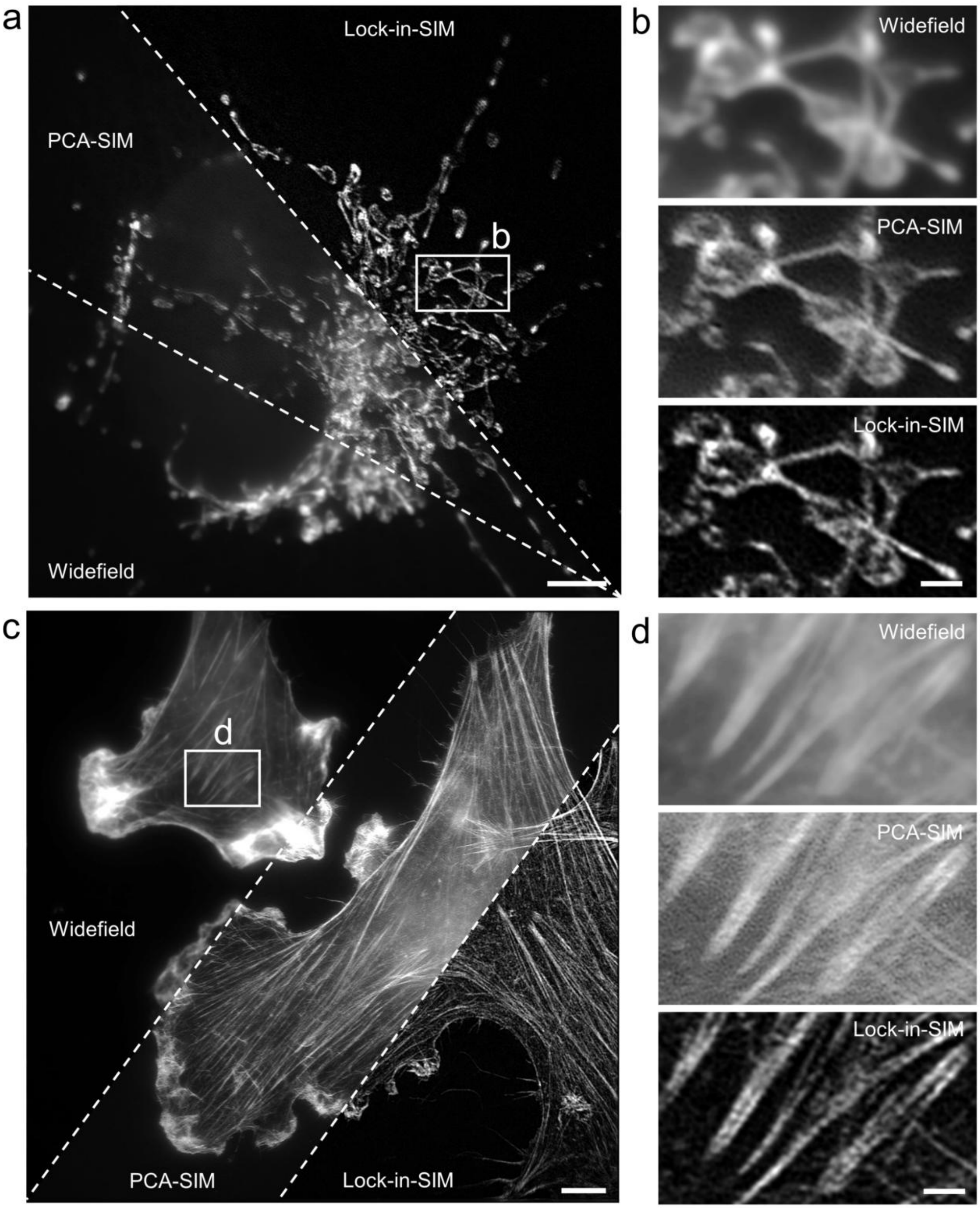

Supplementary Fig. 12. Reconstruction comparison of open-access mitochondria and actin data[20] using different 2D-SIM algorithms. a, Widefield image (bottom) of mitochondria and super-resolution images reconstructed using PCA-SIM (middle) and Lock-in-SIM (right). **b**, Magnified widefield and super-resolution images reconstructed using different methods of the white boxed regions in **a**. **c**, Widefield image (left) of actin and super-resolution images reconstructed using PCA-SIM (middle) and Lock-in-SIM (right). **d**, Magnified widefield and super-resolution images reconstructed using different methods of the white boxed regions in **c**. The comparison highlights that Lock-in-SIM provides superior background suppression and ultrastructure resolution. Scale bars, 5 μm (**a,c**) and 1 μm (**b,d**).

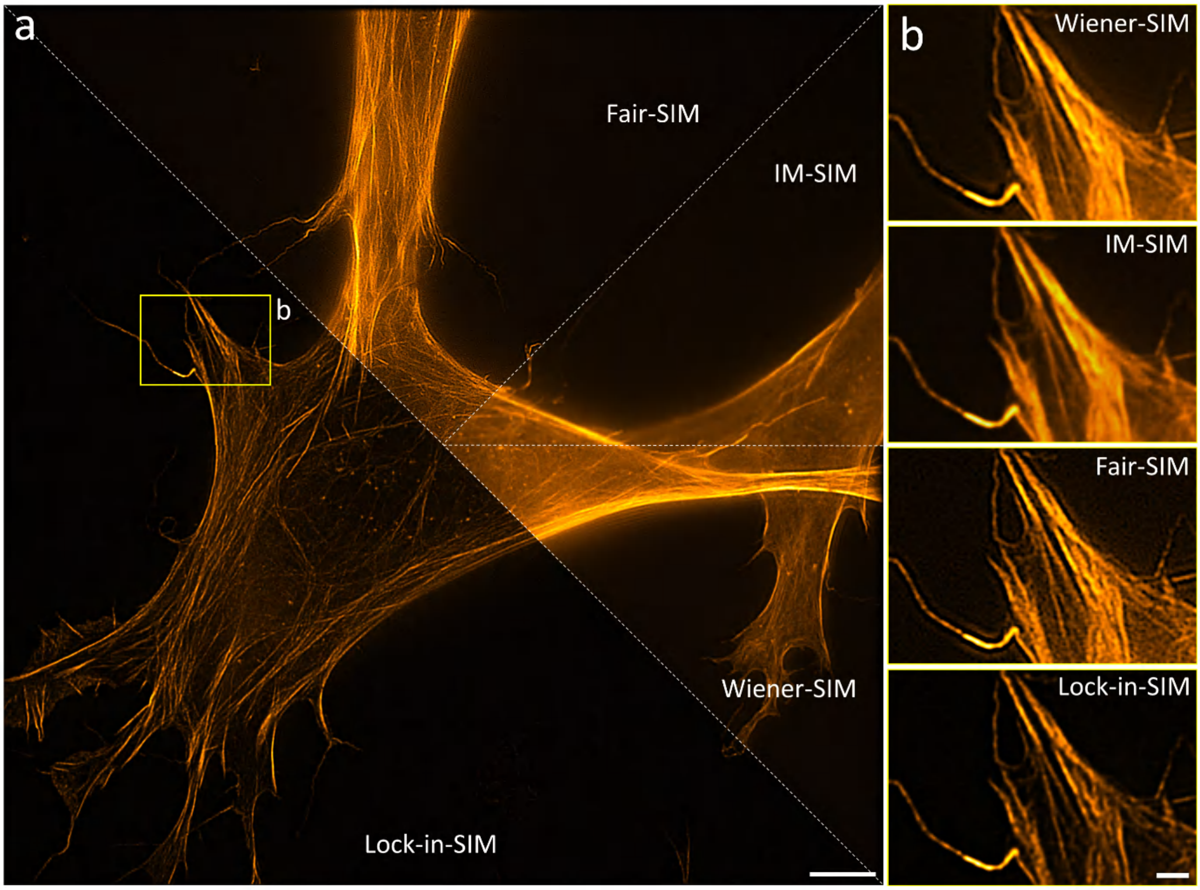

Supplementary Fig. 13. Reconstruction comparison of open-access actin data[21] using different 2D-SIM algorithms. a, Super-resolution images reconstructed using Wiener-SIM, IM-SIM, Fair-SIM, and Lock-in-SIM. **b**, Magnified images of the orange boxed region in **a**. Scale bars, 5 μm (**a**) and 1 μm (**b**).

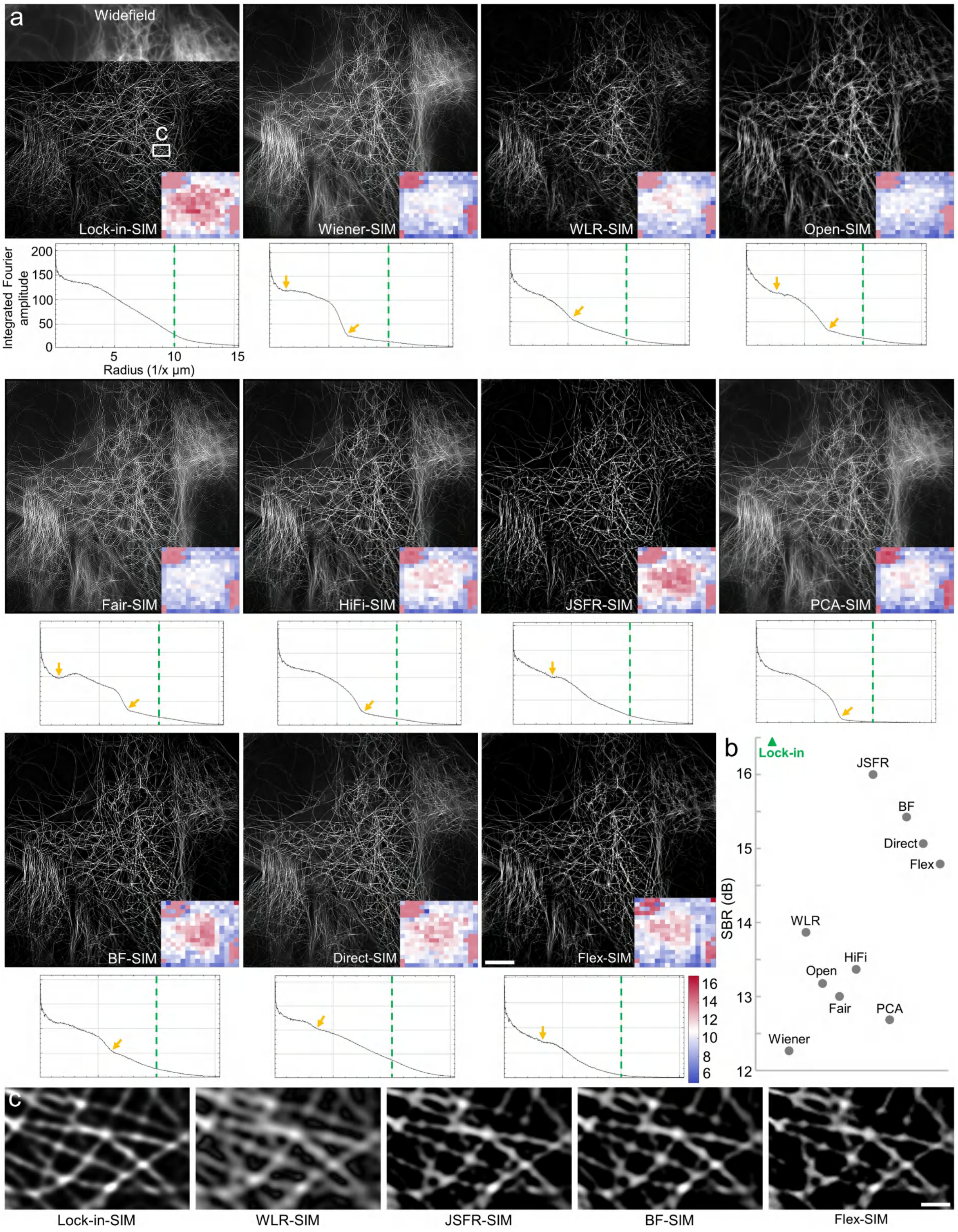

Supplementary Fig. 14. Reconstruction comparison of Tubulin-488-labeled high-background microtubules using different 2D-SIM algorithms. a, First, third and fifth rows: widefield and super-resolution reconstructed image of microtubules reconstructed using Lock-in-SIM, Wiener-SIM, WLR-SIM, Open-SIM, Fair-SIM, HiFi-SIM, JSFR-SIM, PCA-SIM, BF-SIM, Direct-SIM, and Flex-SIM. Bottom-right insets show the corresponding local SBR maps, with red and blue denoting higher and lower SBR, respectively. Second, fourth and sixth rows: the

corresponding integrated Fourier amplitude profiles plotted along the radial direction of the Fourier transformed super-resolution images. The x- and y- coordinates of all plots are the same. Orange arrows indicate the abnormal frequency drop-off, corresponding to the possible image artifacts in the spatial domain. Green dashed lines indicate the frequency points corresponding to ~100-nm resolution. **b**, SBR values of super-resolution images reconstructed by different algorithms in **a**. **c**, Magnified images of the white boxed region in **a**, showing a local comparison of the high-SBR results from Lock-in-SIM, WLR-SIM, JSFR-SIM, BF-SIM, and Flex-SIM. Among them, WLR-SIM exhibits ringing-like artifacts and lower effective image resolution, while JSFR-SIM, BF-SIM, and Flex-SIM display severe loss of structure information. By contrast, Lock-in-SIM simultaneously provides superior background suppression and image fidelity (i.e, structural integrity and ultrastructure preservation). Scale bar, 5 μm (**a**) and 0.5 μm (**c**).

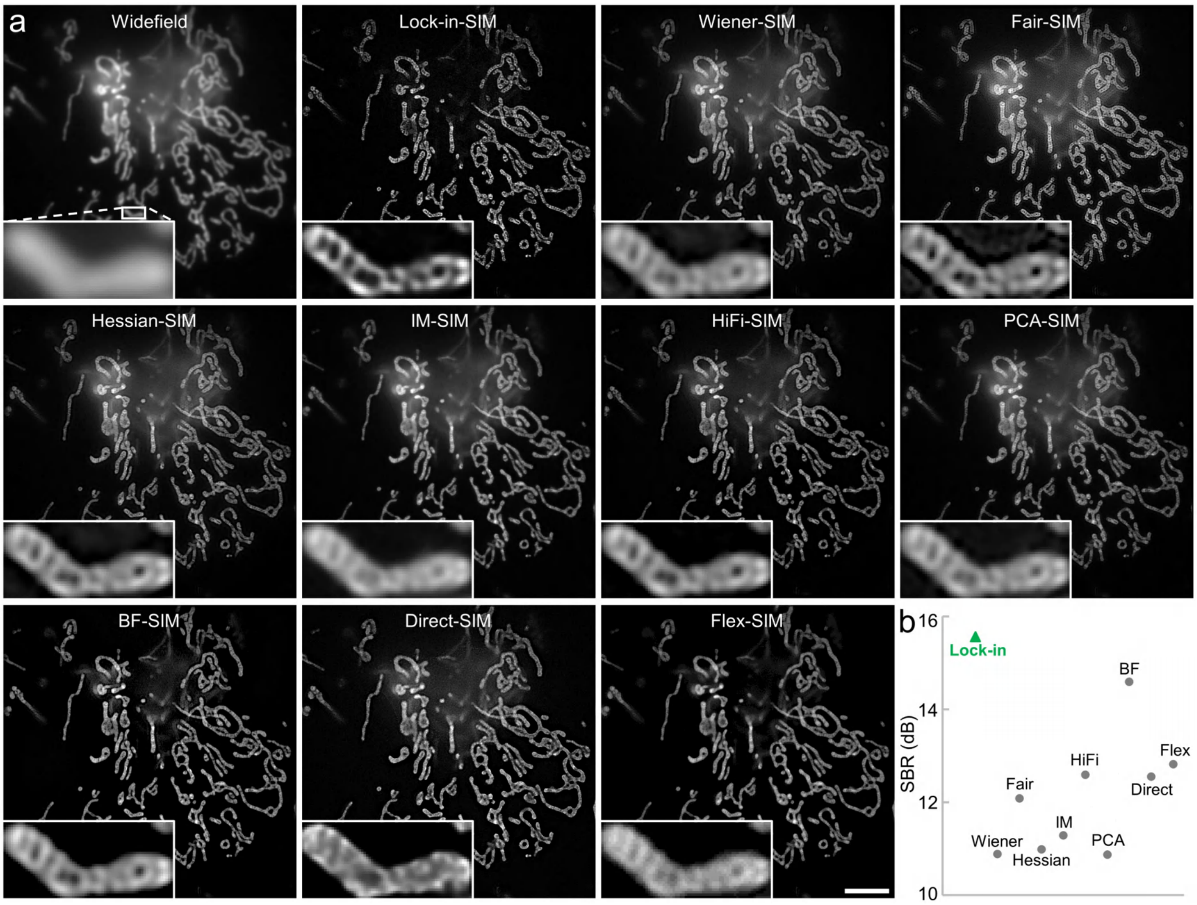

Supplementary Fig. 15. Reconstruction comparison of PKmito DEEP RED-labeled mitochondria inner membrane using different 2D-SIM algorithms. a, Widefield image of mitochondria and super-resolution images reconstructed using Lock-in-SIM, Wiener-SIM, Fair-SIM, Hessian-SIM, IM-SIM, HiFi-SIM, PCA-SIM, BF-SIM, Direct-SIM, and Flex-SIM. Insets show the magnified views of the white-boxed region. **b**, SBR values of super-resolution images reconstructed by different algorithms in **a**. The comparison highlights that Lock-in-SIM provides superior background suppression and membrane resolving power. Scale bar, 5 μm (**a**).

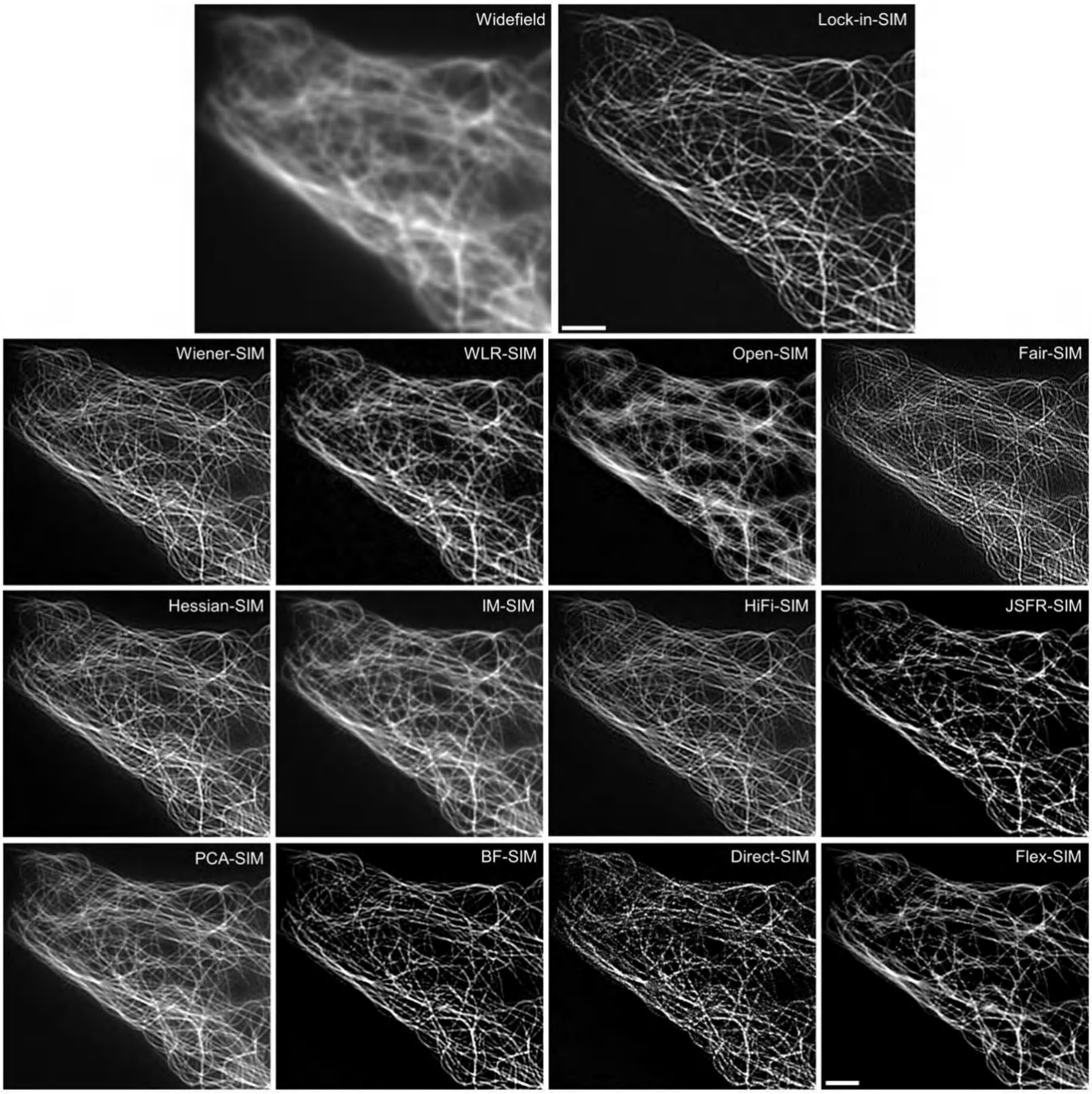

Supplementary Fig. 16. Reconstruction comparison of Tubulin-488-labeled dense microtubules using different 2D-SIM algorithms. Widefield image of microtubules and corresponding super-resolution images reconstructed using Lock-in-SIM, Wiener-SIM, WLR-SIM, Open-SIM, Fair-SIM, Hessian-SIM, IM-SIM, HiFi-SIM, JSFR-SIM, PCA-SIM, BF-SIM, Direct-SIM, and Flex-SIM, demonstrating superior background suppression, structural integrity, ultrastructure preservation, and image fidelity of Lock-in-SIM. Scale bar, 2 μm.

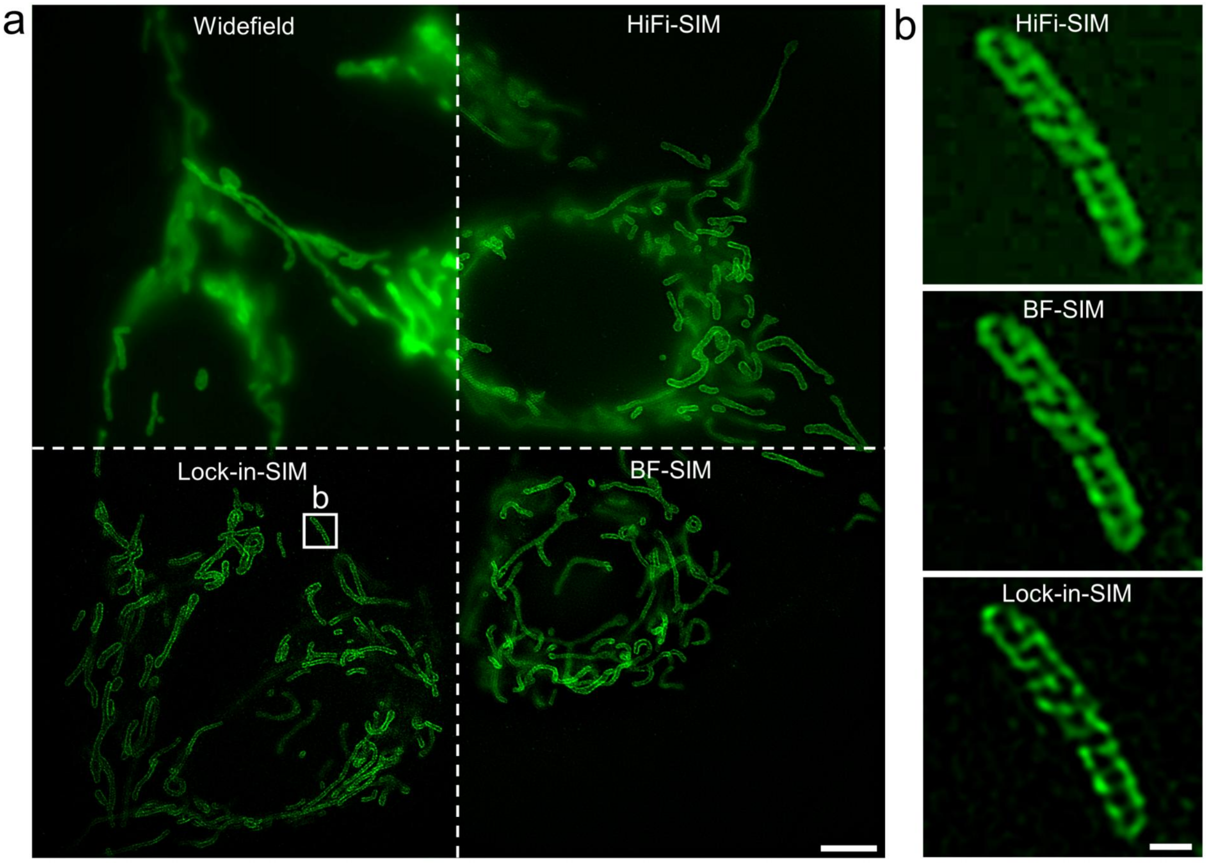

Supplementary Fig. 17. Reconstruction comparison of mitochondrial structure using different 2D-SIM algorithms. a, Widefield image and corresponding super-resolution images reconstructed using HiFi-SIM, BF-SIM, and Lock-in-SIM. **b**, Magnified images of the white boxed regions in **a**. Scale bars, 4 μm (**a**) and 0.4 μm (**b**).

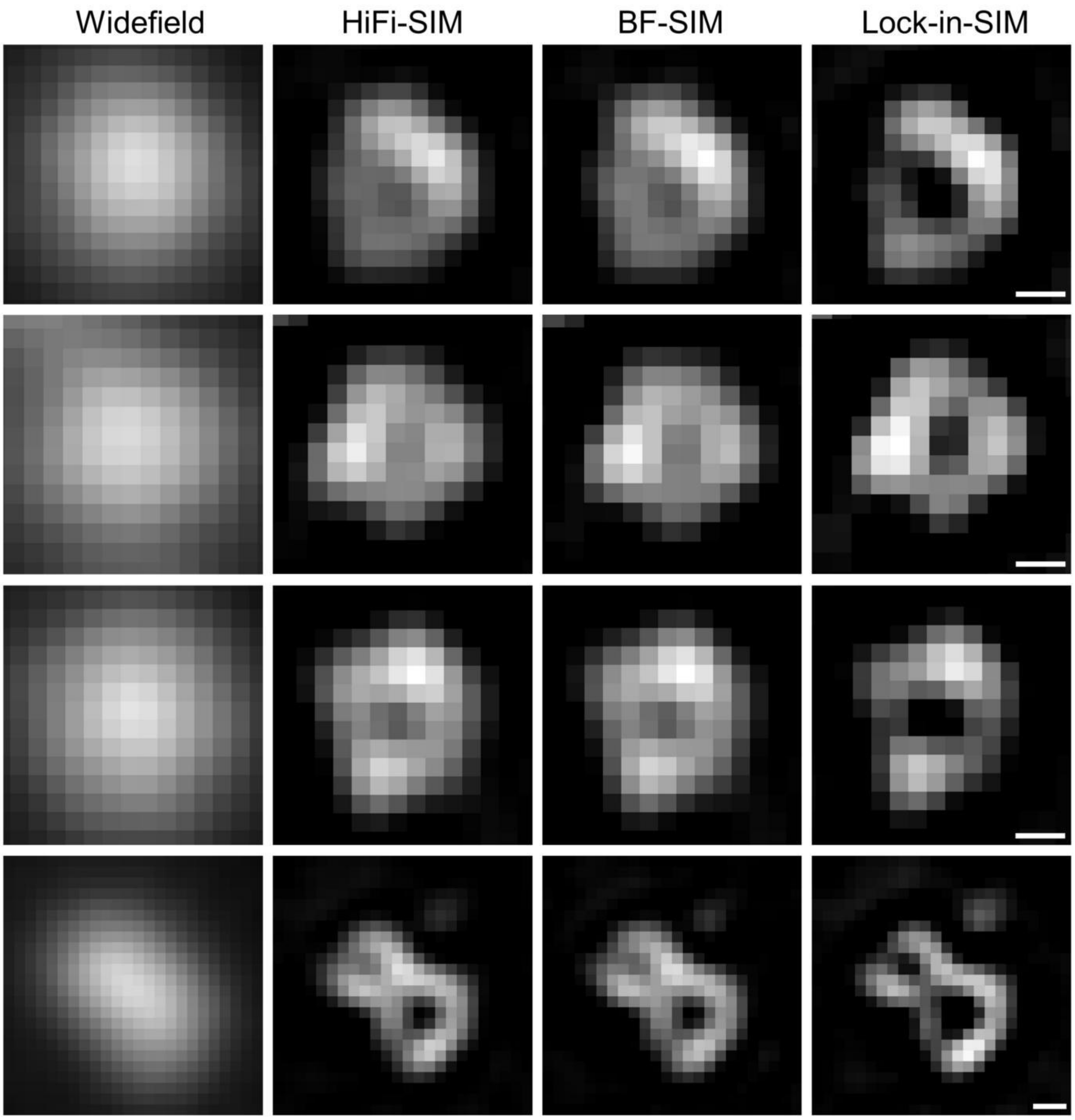

Supplementary Fig. 18. Reconstruction comparison of clathrin-coated pits data using different 2D-SIM algorithms. Widefield images and corresponding super-resolution images reconstructed using HiFi-SIM, BF-SIM, and Lock-in-SIM. Scale bars, 0.1 μm.

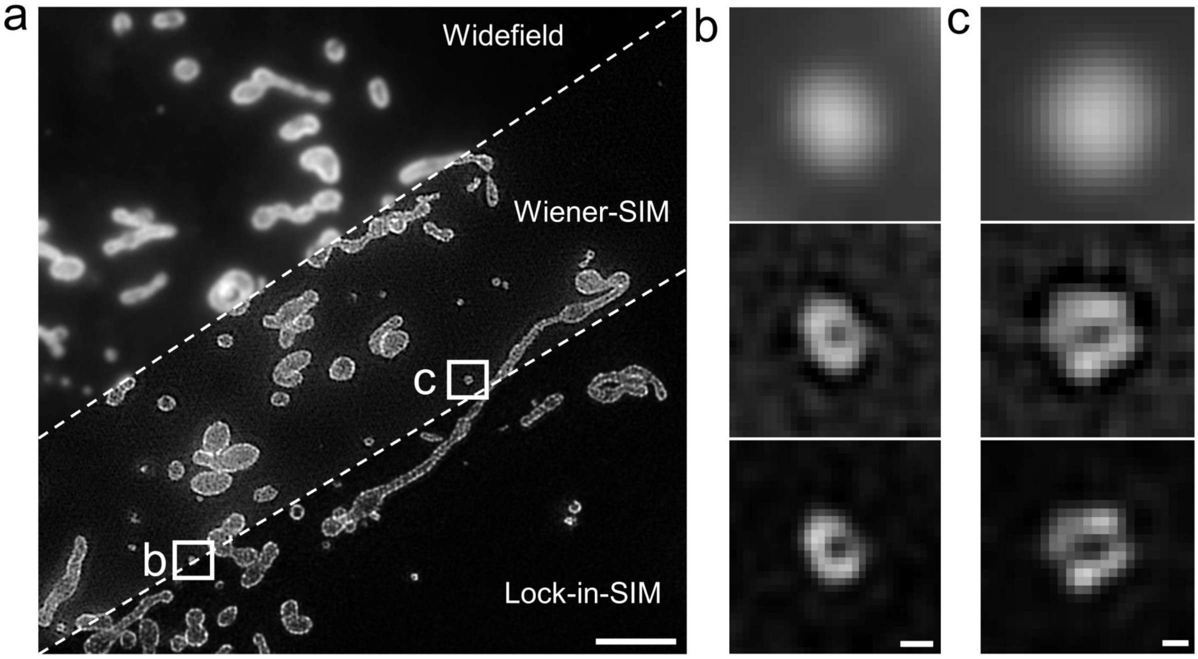

Supplementary Fig. 19. Reconstruction comparison of Tomm20-AF488-labeled mitochondria and mitochondrial-derived vesicles. a, Widefield image and corresponding super-resolution images reconstructed using Wiener-SIM and Lock-in-SIM. **b**,**c** Magnified images of the white boxed mitochondrial-derived vesicles in **a**. From top to bottom, Widefield, Wiener-SIM, and Lock-in-SIM. Scale bars, 3 μm (**a**) and 0.1 μm (**b,c**).

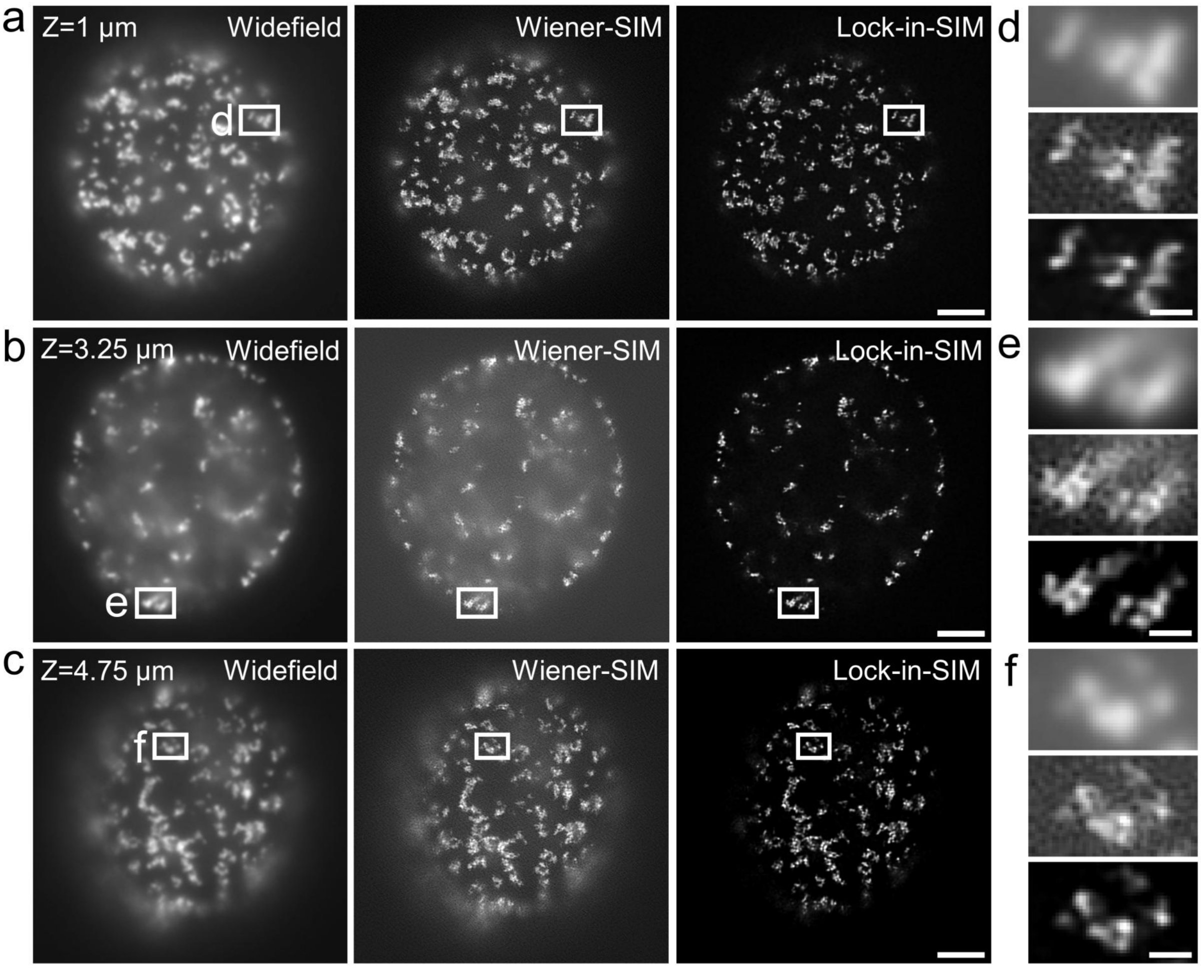

Supplementary Fig. 20. Optical-sectioning reconstruction comparison of EdU replication-labelled nuclei. a-c, Widefield images and corresponding super-resolution images reconstructed using Wiener-SIM and Lock-in-SIM of the same sample at different sample depths. **d-f**, Magnified images of the white boxed regions in **a-c**. From top to bottom, Widefield, Wiener-SIM, and Lock-in-SIM. Scale bars, 3 μm (**a-c**) and 0.5 μm (**d-f**).

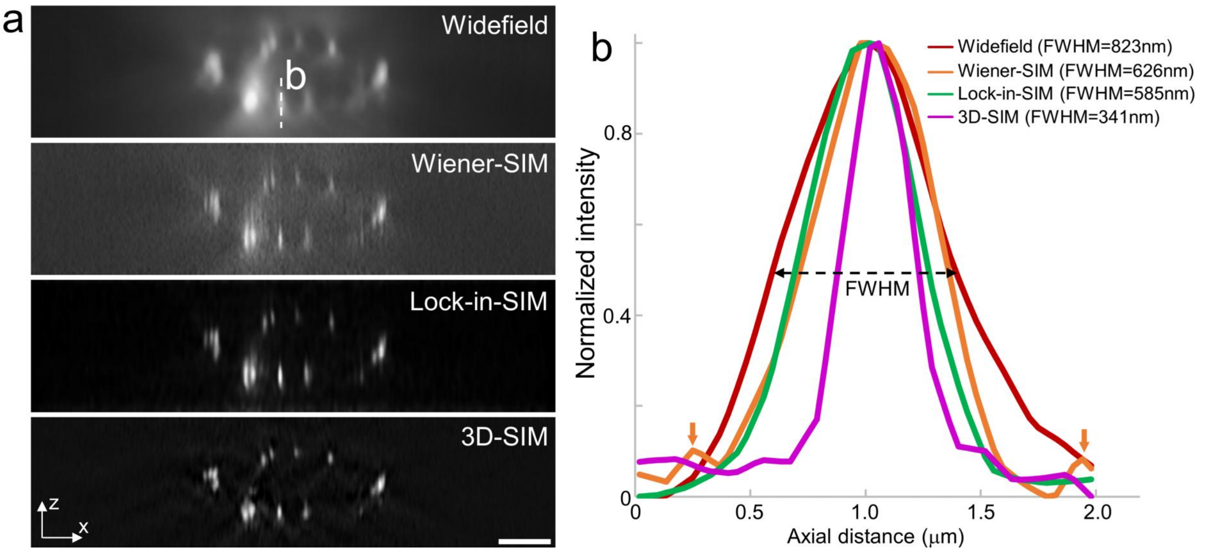

Supplementary Fig. 21. 3D reconstruction comparison of EdU replication-labelled nuclei. a, Axial views of the widefield images and corresponding super-resolution images reconstructed using Wiener-SIM, Lock-in-SIM, and 3D-SIM of the same EdU sample in Supplementary Fig. 17. **b**, Normalized intensity profiles along the dashed line region in **a** and the corresponding axial full width at half maximum (FWHM) values. Orange arrows indicate the background artifacts in Wiener-SIM image. Scale bars, 2 μm (**a**).

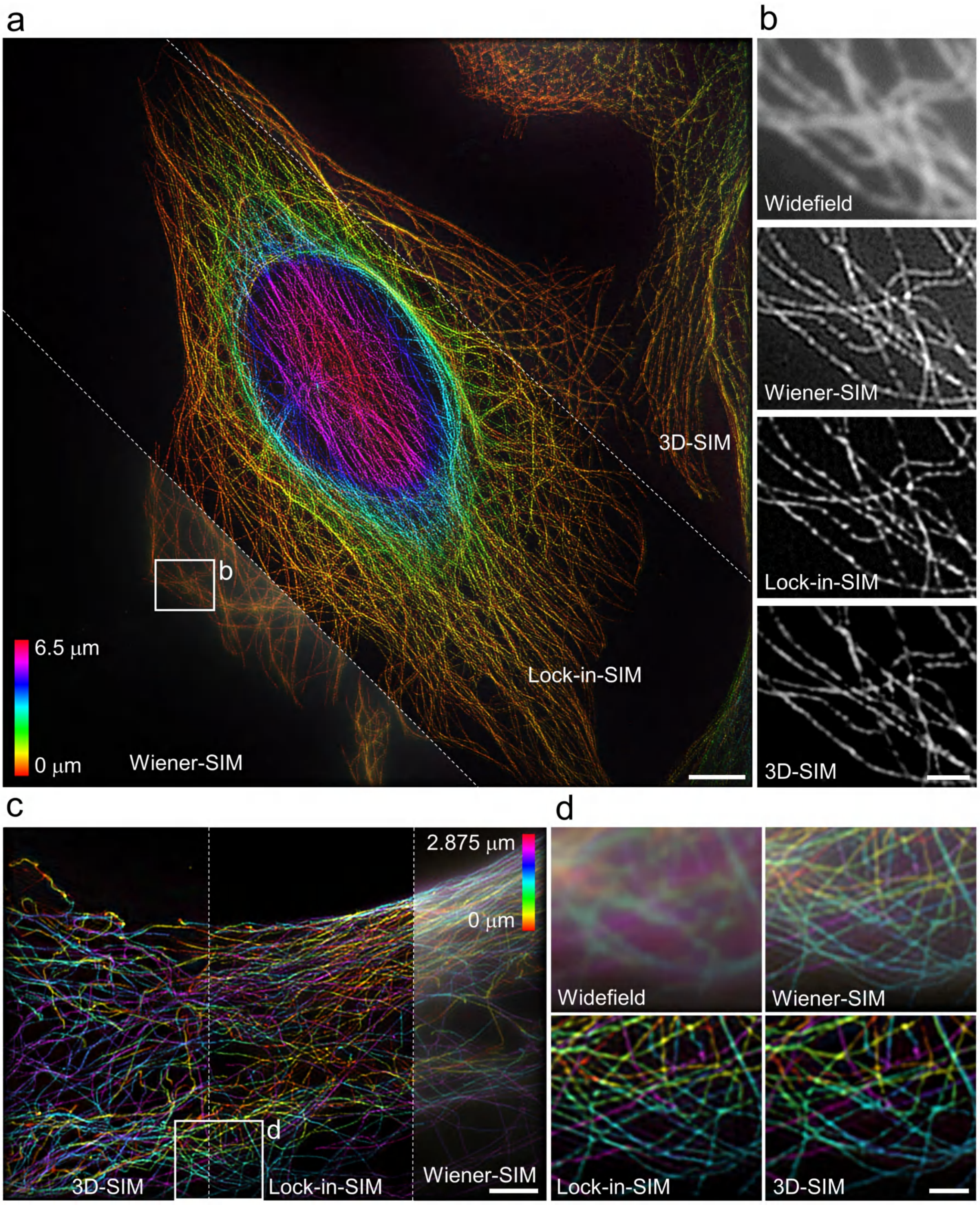

Supplementary Fig. 22. Reconstruction comparison of Lock-in SIM, Wiener-SIM, and 3D-SIM. a, Depth-coded maximum intensity projection of the anti-tubulin-labeled microtubule stack reconstructed using Lock-in-SIM (middle), 3D-SIM (middle right), and Wiener-SIM (bottom left). **b**, Magnified images of the white boxed region in **a**. **c**, Depth-coded maximum intensity projection of the anti-tubulin-labeled microtubule stack reconstructed using Lock-in-SIM (middle), 3D-SIM (left), and Wiener-SIM (right). **d**, Magnified images of the white boxed region in **c**. Scale bars, 5 μm (**a**), 1 μm (**b,d**), and 3 μm (**c**).

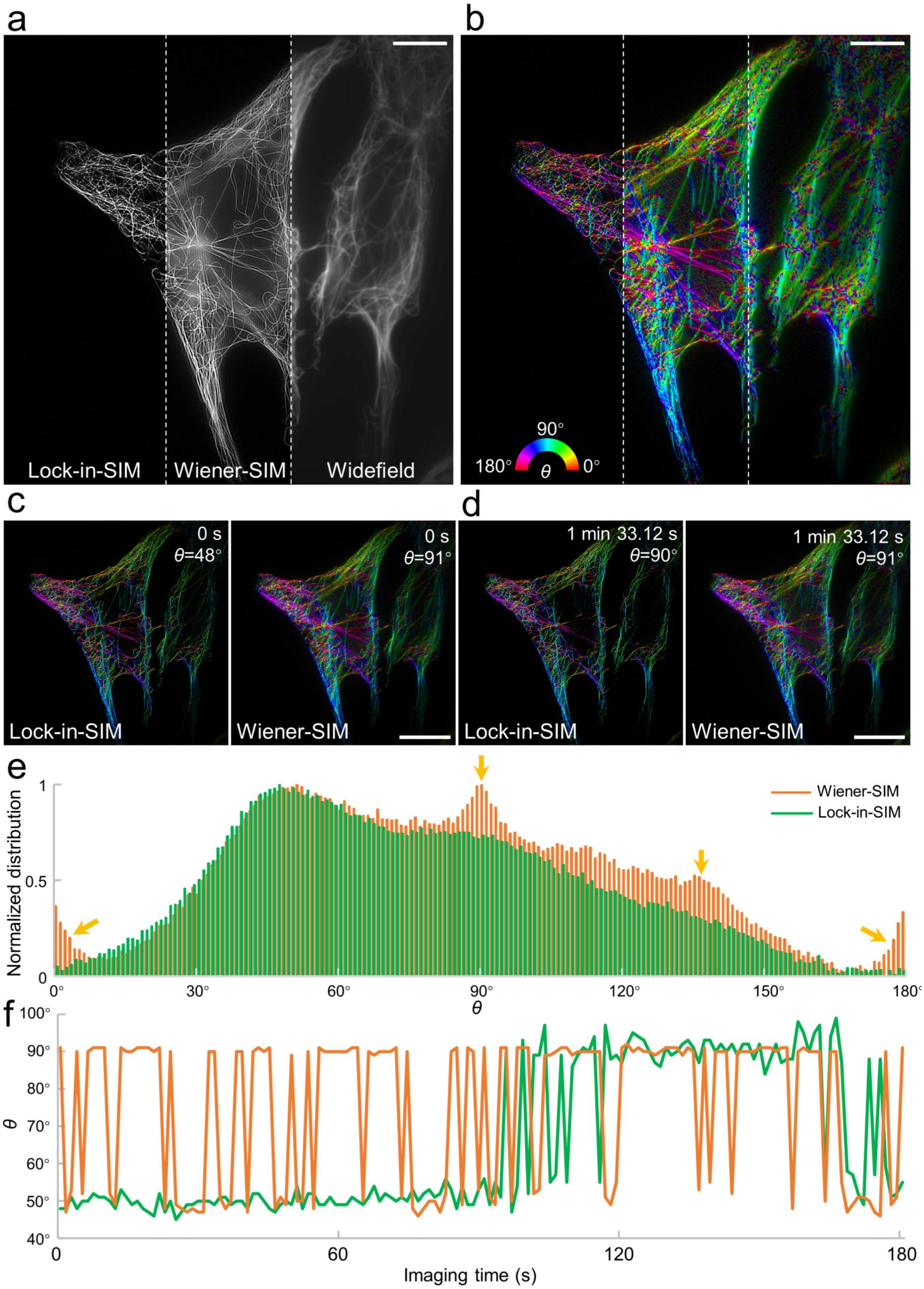

Supplementary Fig. 23. Quantitative orientation analysis of living microtubules. a, Widefield image (right) of Tubulin-488-labeled living microtubule and super-resolution images reconstructed using Wiener-SIM (middle) and Lock-in-SIM (left). **b**, Orientation distributions of

the reconstructed Lock-in-SIM (left), Wiener-SIM (middle) and Widefield (right) images in **a**. **c,d**, Orientation distributions of the reconstructed Lock-in-SIM (left) and Wiener-SIM (right) images in **a** at timepoints of t=0 s (**c**) and t=1 min 33.12 s (**d**). **e**, Normalized orientation distribution histograms of the Wiener-SIM (orange) and Lock-in-SIM (green) images in **c**. Orange arrows indicate the abnormal values of θ in the Wiener-SIM result due to the influence of high background. **f**, Time-lapse Lock-in-SIM (green) and Wiener-SIM (orange) θ fluctuations, showing the analysis error of the Wiener-SIM results (sharp fluctuation of the orange curve) and the change in microtubule spatial organization (green curve). Scale bars, 8 μm (**a,b**) and 15 μm (**c,d**).

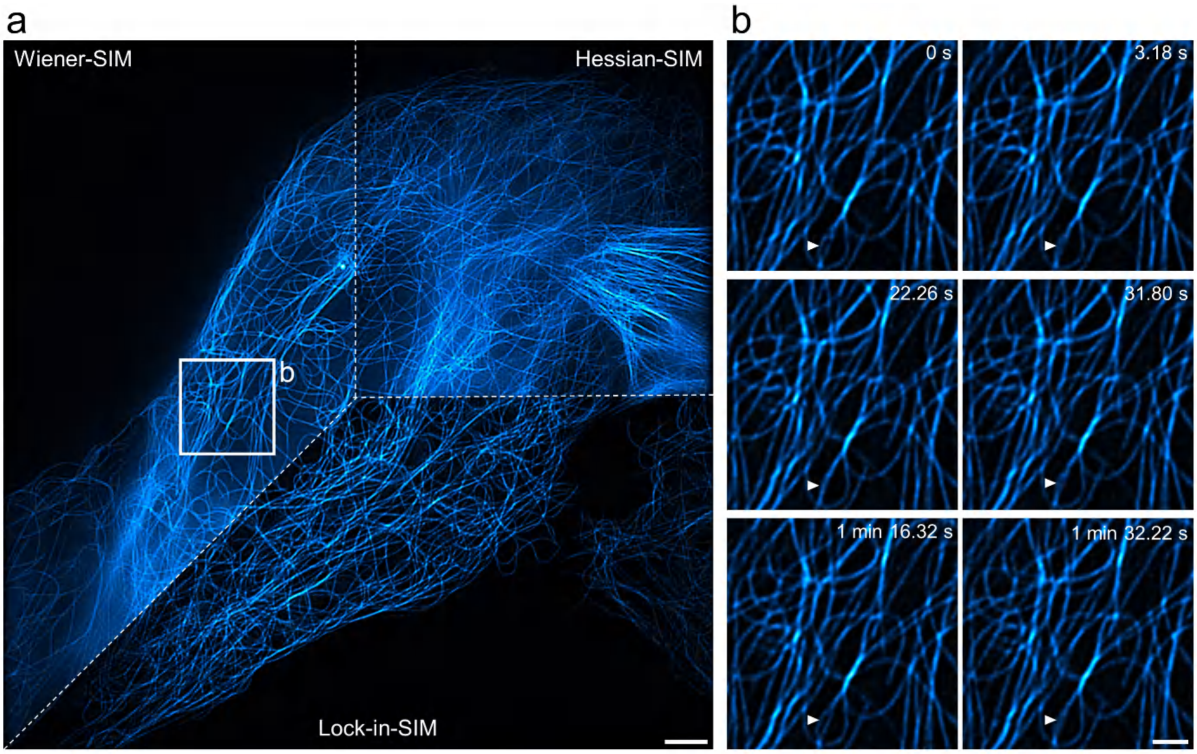

Supplementary Fig. 24. Live-cell imaging of the tubulin-SiR-labeled microtubules. a, Super-resolution images of microtubules reconstructed using Wiener-SIM (left), Hessian-SIM (top right), and Lock-in-SIM (bottom right). **b**, Magnified time-lapse images of the white boxed region in **a**. White arrowheads indicate the contact and separation of two tubules. Scale bars, 3 μm (**a**) and 1 μm (**b**).

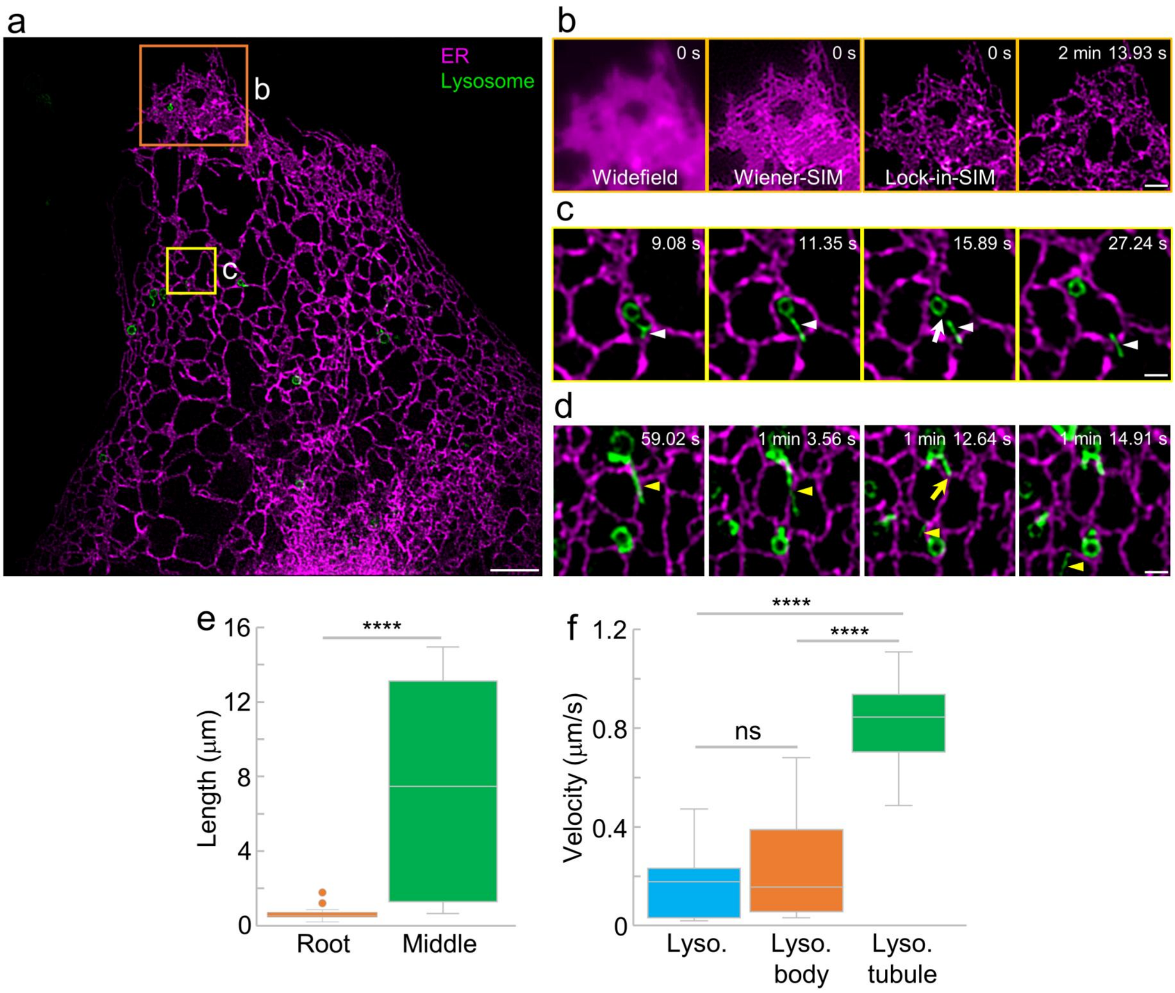

Supplementary Fig. 25. Live-cell imaging of ER and lysosome ultrastructures, dynamics, and interactions using Lock-in-SIM. **a**, Two-color Lock-in-SIM image of Sec61β-DY549-labeled living ER (magenta) and Rab7-AF488-labeled lysosome (green). **b**, Widefield (left), Wiener-SIM (middle left), and time-lapse Lock-in-SIM (middle right and right) images of the orange boxed ER matrix region in **a**. Only the ER channel is shown. **c**, Time-lapse Lock-in-SIM images of the yellow boxed regions in **a**. White arrowheads indicate the ER-mediated lysosome tubule fission event. White arrow indicates the fission position (at the root of the lysosome tubule). **d**, Time-lapse Lock-in-SIM images of another ER-mediated lysosome tubule fission event (yellow arrowheads). The yellow arrow indicates the fission position (in the middle of the lysosome tubule). **e**, Statistical boxplot of the lysosome tubule length when ER-mediated lysosome tubule fission occurs at the root (orange) or middle (green) positions (n=23 for the root group and n=10 for the middle group). **f**, Statistical boxplot of the velocities of the integral lysosome before fission (blue), lysosome without tubule after fission (orange), and lysosome with only tubules after fission (green) (n=15 per group). ns $P \geq 0.05$ (no statistical difference), **** $P<0.0001$ (extremely significant statistical difference). The middle line in the box corresponds to the mean value. The upper and lower boundary lines correspond to the 15th and 75th percentiles, respectively. The whiskers represent the min and max values. Scale bars, 3 μm (**a**), 1 μm (**b**), and 0.5 μm (**c,d**).

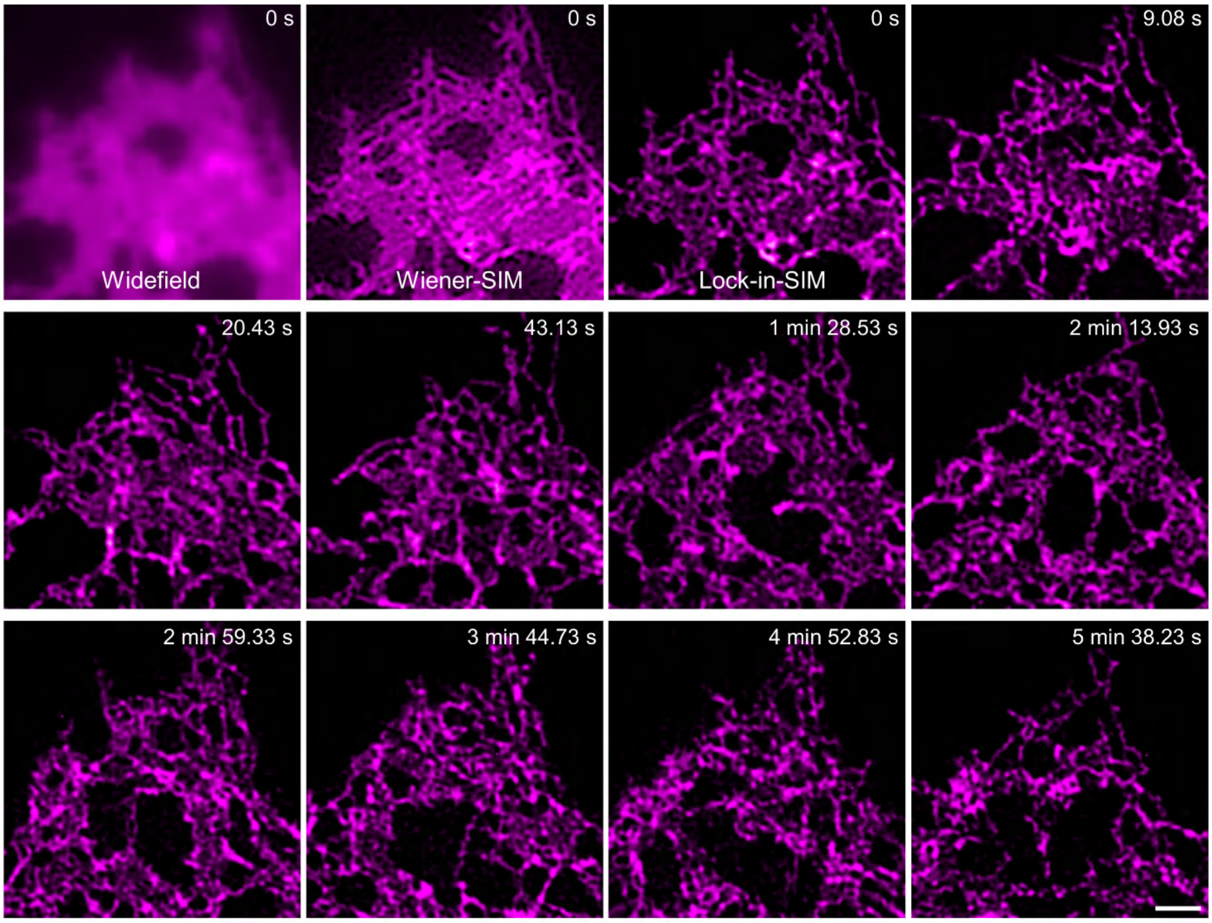

Supplementary Fig. 26. Live-cell imaging of Sec61β-DY549-labeled ER. Widefield (left, first column), Wiener-SIM (middle left, first column), and time-lapse Lock-in-SIM images of different ER regions showing the high dynamics of the ER matrix. Scale bar, 3 μm.

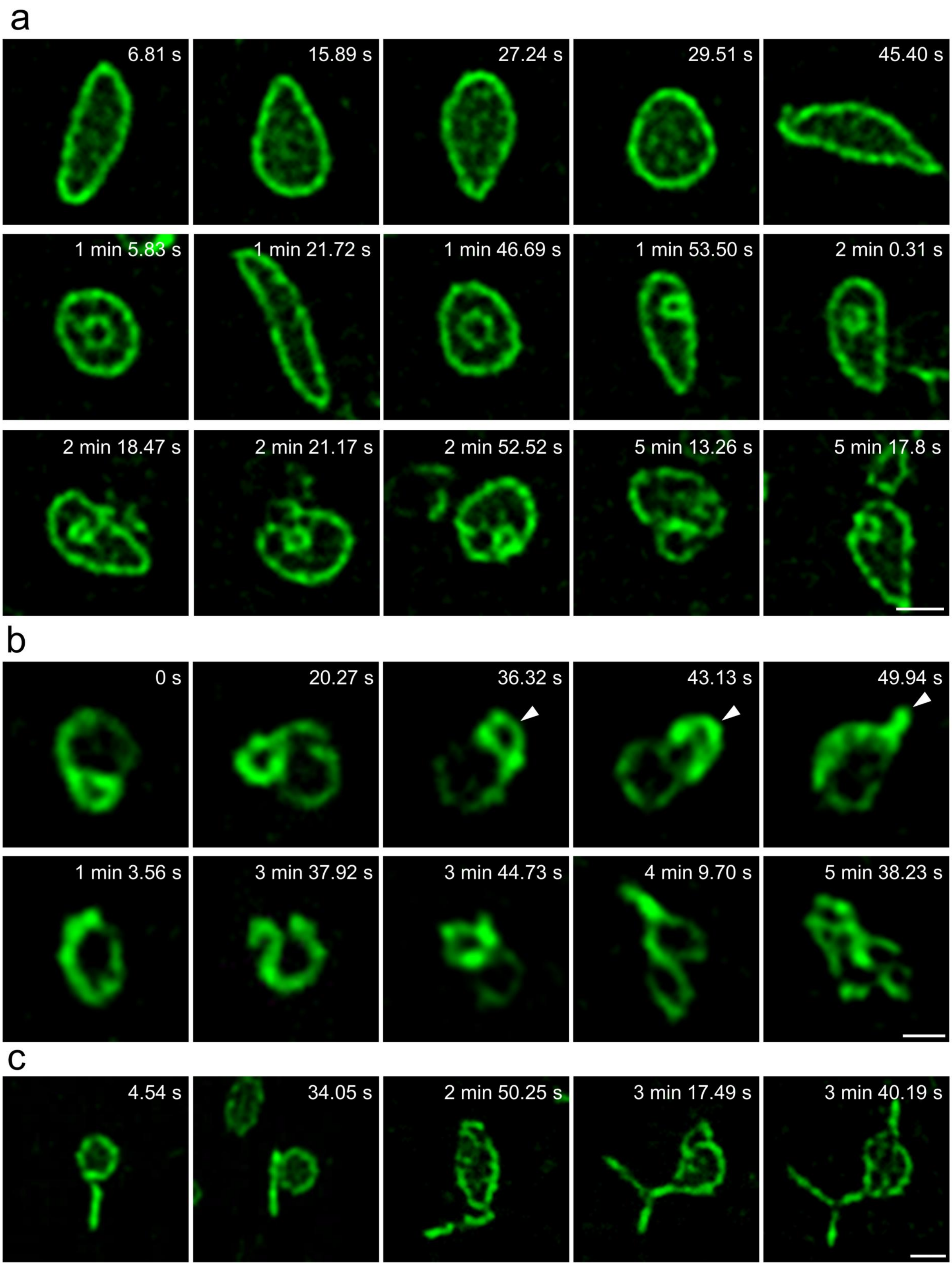

Supplementary Fig. 27. Live-cell Lock-in-SIM imaging of Rab7-EGFP/AF488-labeled lysosomes. a-c, Time-lapse Lock-in-SIM images of three typical lysosomes showing drastic changes in the outer and inner morphologies of the lysosomes. White arrowheads in **b** indicate the formation of a lysosome tubule. Scale bars, 0.5 μm (**a**,**c**) and 0.3 μm (**b**).

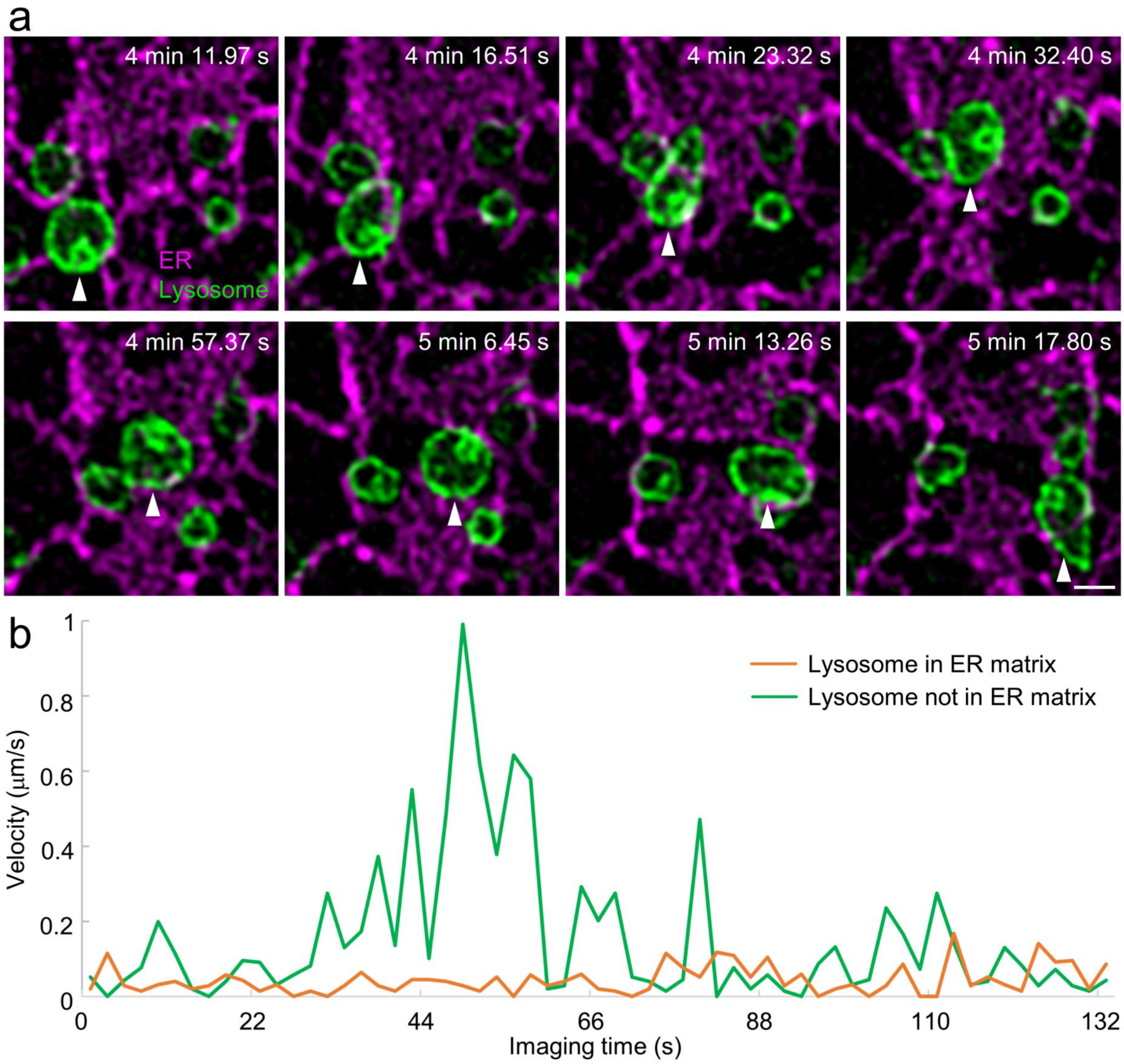

Supplementary Fig. 28. Two-color live-cell Lock-in-SIM imaging of the lysosome movement in the ER matrix. a, Time-lapse images of composed ER (magenta) and lysosomes (green). White arrowheads indicate the moving lysosome before and after entering the ER matrix. **b**, Velocity differences of the moving lysosome inside (orange) and outside (green) the ER matrix at continuous time points. Scale bar, 0.5 μm (**a**).

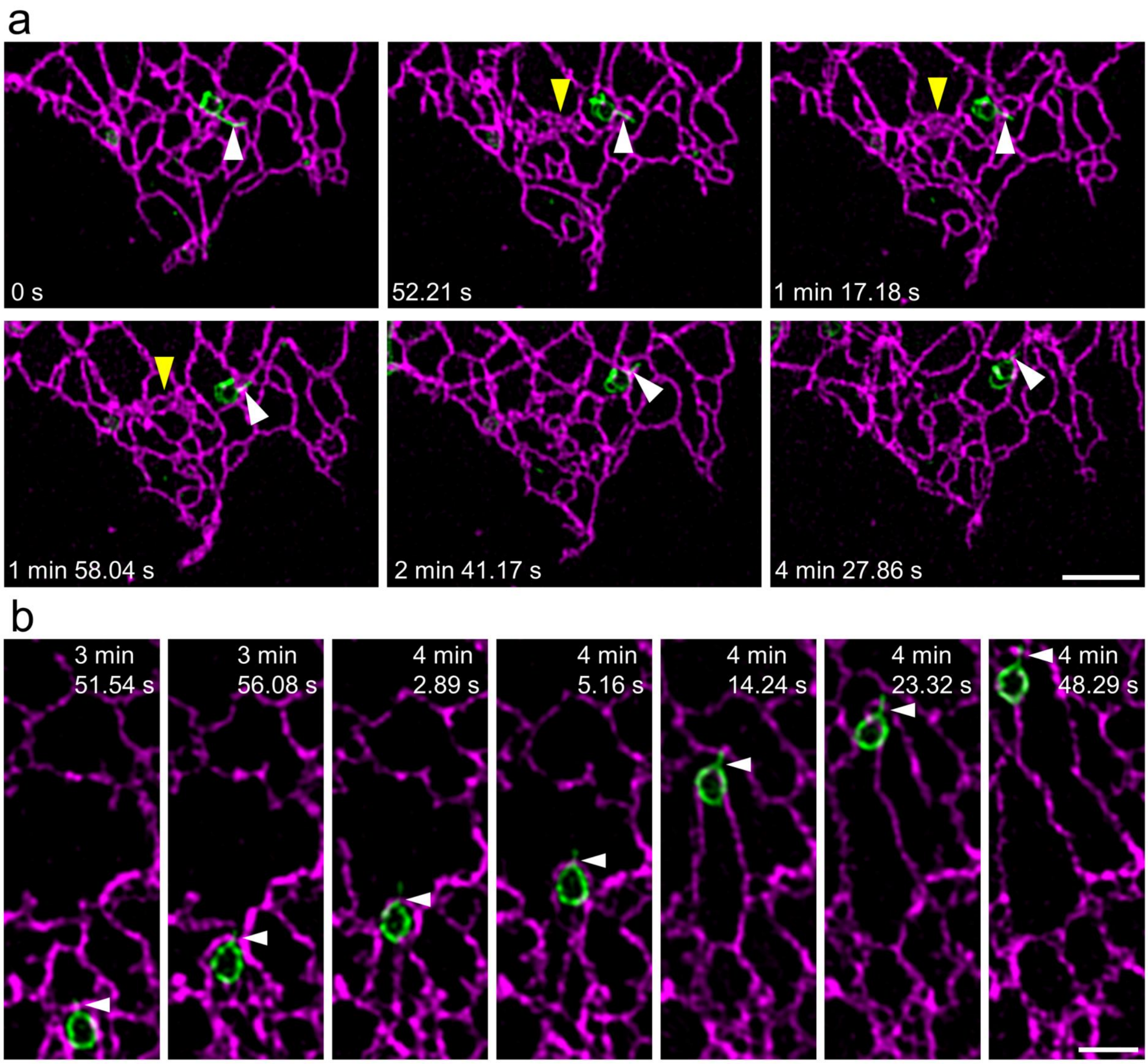

Supplementary Fig. 29. Two-color live-cell Lock-in-SIM imaging of the interaction between lysosomes (green) and the ER (magenta). a, Time-lapse images of the ER (labeled with Sec61β-DY549) and lysosomes (labeled with Rab7-AF488). White arrowheads indicate the lysosome tubule-directed lysosome moving with the intervention of the ER tubule. Yellow arrowheads indicate the ER matrix dynamics. **b**, Time-lapse images of the lysosome tubule-directed lysosome moving with the intervention of the ER tubule (white arrowheads). Scale bars, 2 μm (**a**) and 1 μm (**b**).

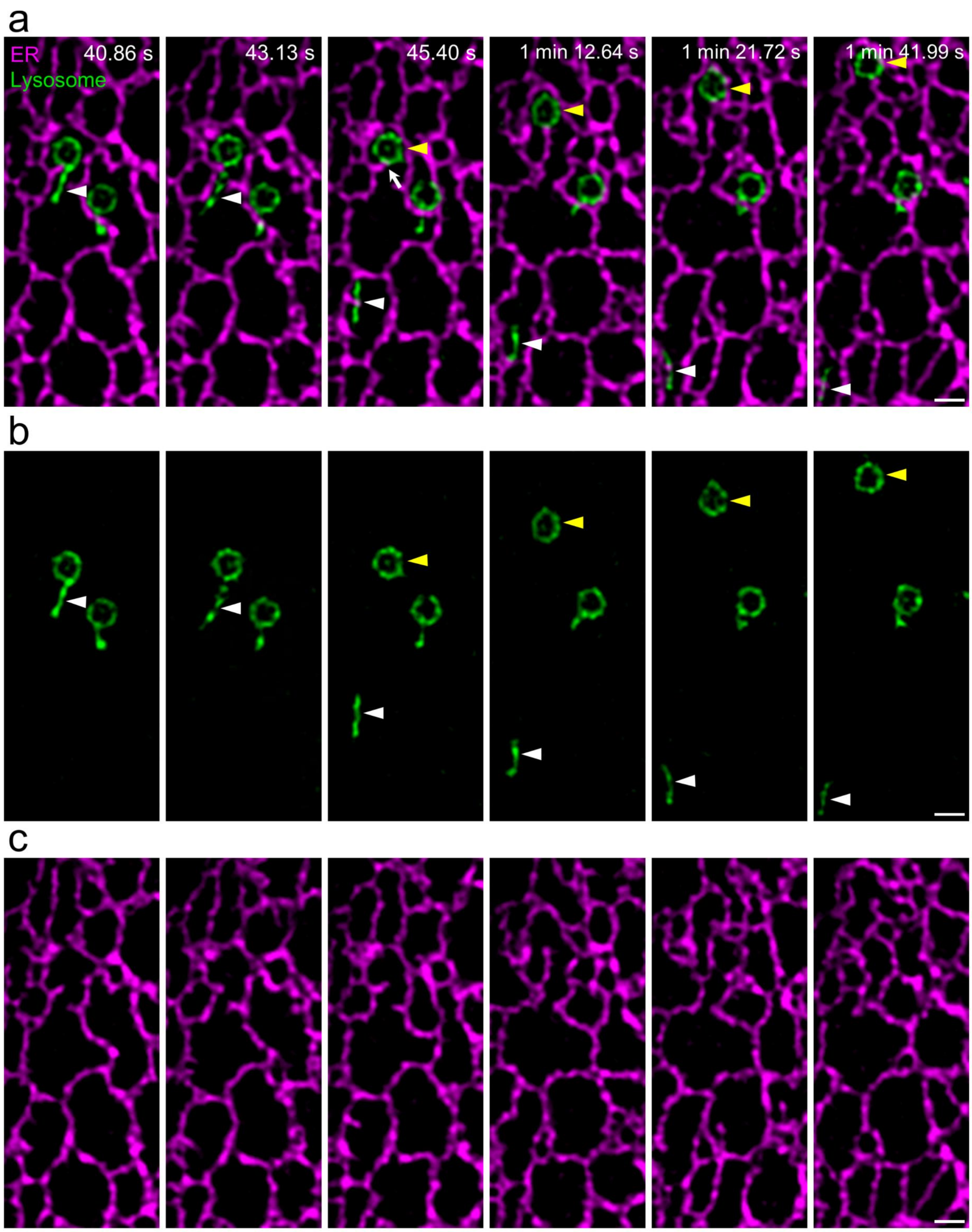

Supplementary Fig. 30. Two-color live-cell Lock-in-SIM imaging of ER-mediated lysosome tubule fission. a-c, Time-lapse images of the ER (labeled with Sec61β-DY549, magenta) and lysosome (labeled with Rab7-AF488, green) (**a**), and single-channel lysosomes (**b**) and the ER (**c**). White arrowheads indicate the moving lysosome tubule before and after fission. The white arrow indicates the fission position. Yellow arrowheads indicate the moving lysosome without tubule after fission. Scale bars, 0.5 μm (**a-c**).

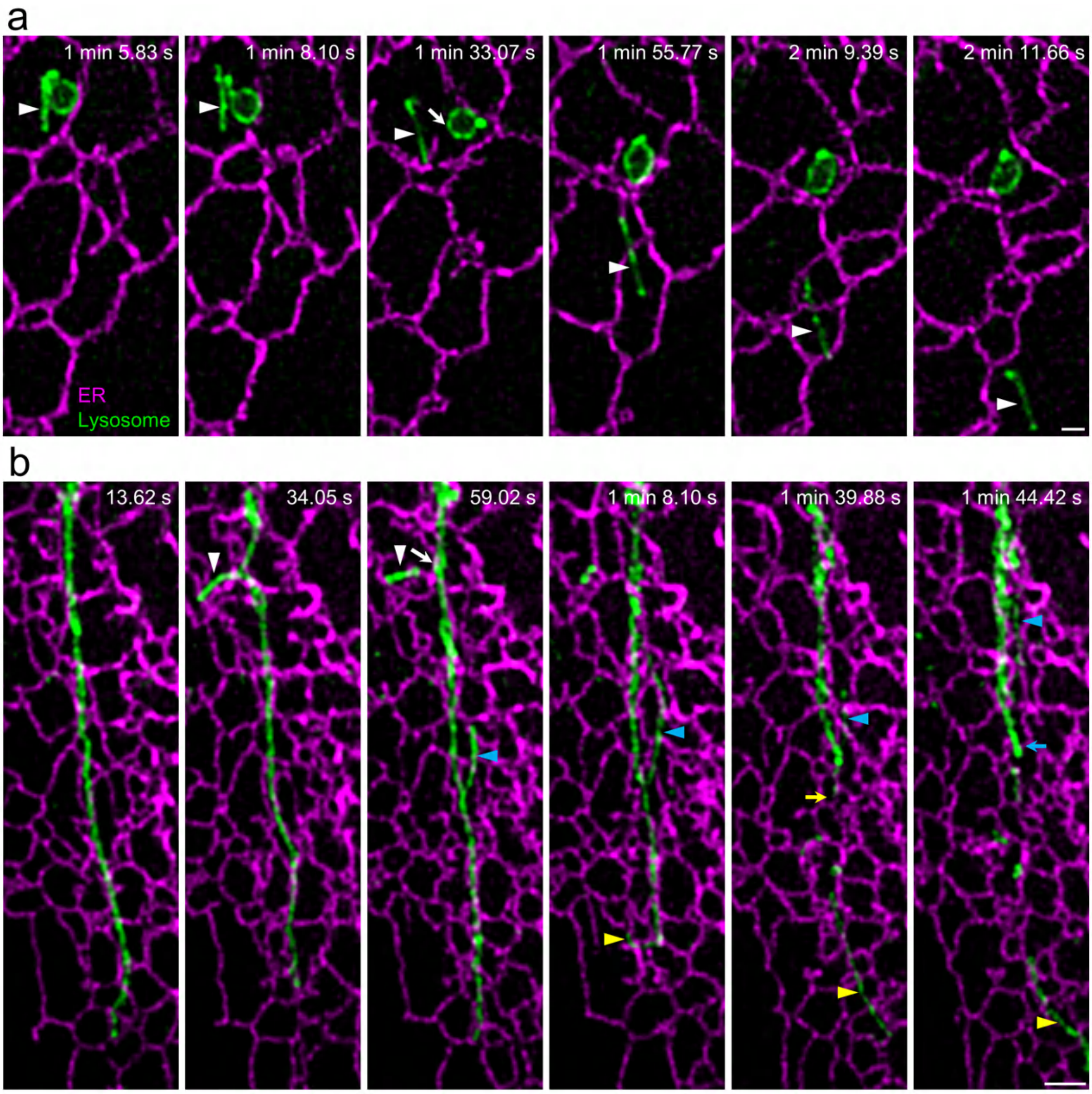

Supplementary Fig. 31. Two-color live-cell Lock-in-SIM imaging of the ER-mediated lysosome tubule fission. a,b, Time-lapse images of the ER (magenta) and lysosomes (green). Colored arrowheads indicate multiple cases of ER-mediated lysosome tubule fission. Colored arrows indicate the fission positions (at the lysosome tubule root in **a** and in the middle in **b**). Scale bars, 0.5 μm (**a**) and 1 μm (**b**).

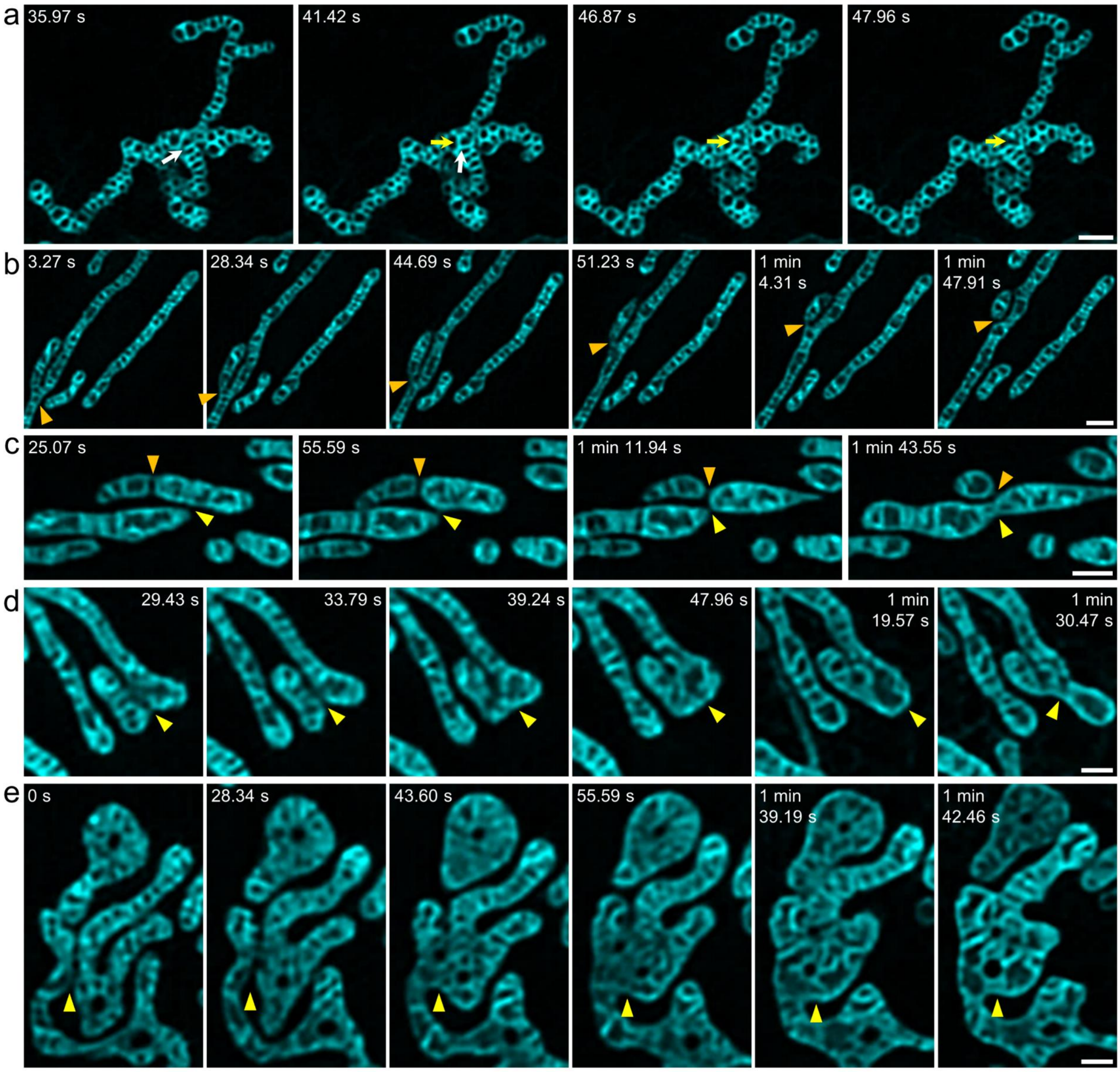

Supplementary Fig. 32. Live-cell Lock-in-SIM imaging of PKmito DEEP RED-labeled mitochondria and inner membrane dynamics. White and yellow arrows in **a** indicate cases of mitochondria inner membrane fusion and fission, respectively. Orange and yellow arrowheads in **b-e** indicate cases of mitochondrial fission and fusion, respectively. Scale bars, 1 μm (**a-c**) and 0.6 μm (**d,e**).

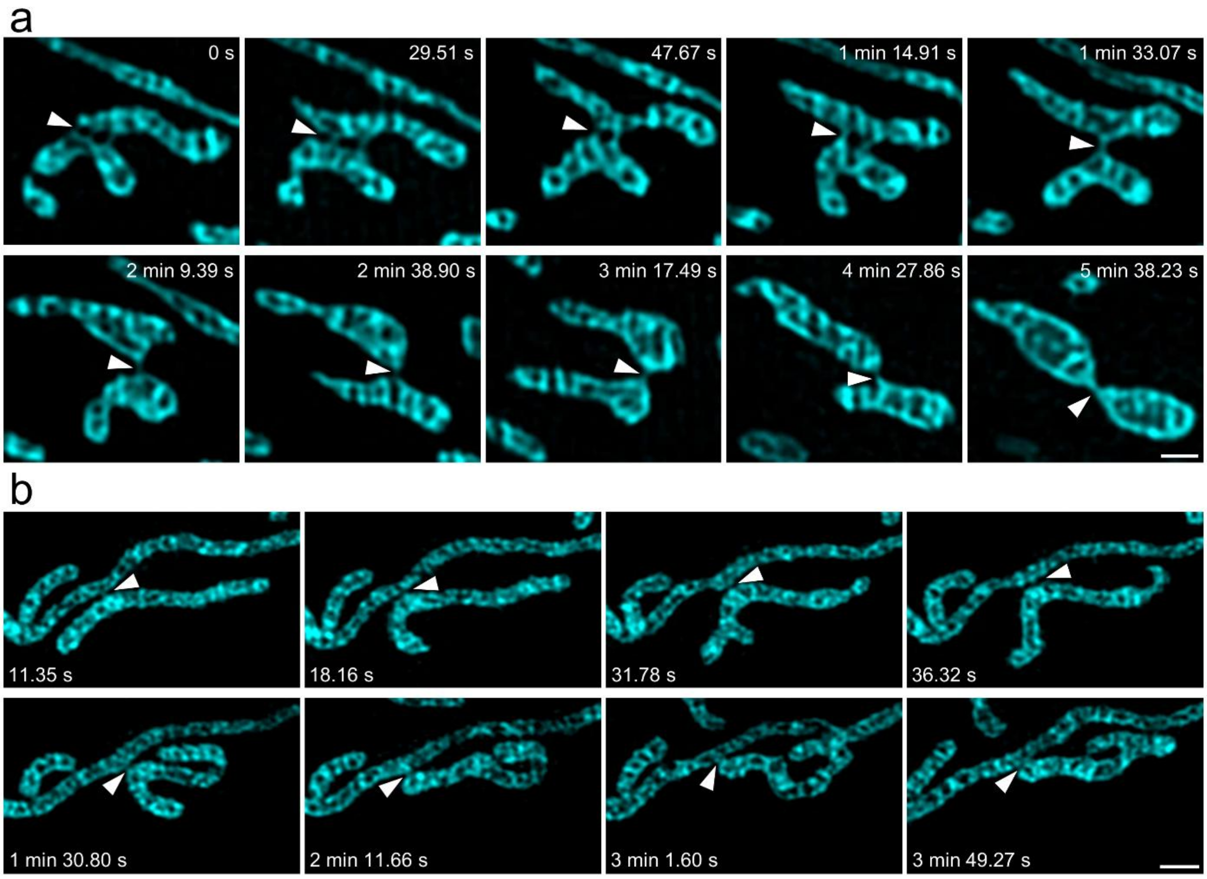

Supplementary Fig. 33. Live-cell Lock-in-SIM imaging of the inner membranes of PKmito DEEP RED-labeled living mitochondria. a,b, Time-lapse Lock-in-SIM images of mitochondrial dynamics in different sample regions showing the behavior such as the membrane connections between mitochondria (white arrowheads) and cristae dynamics. Scale bars, 0.5 μm (**a**) and 1 μm (**b**).

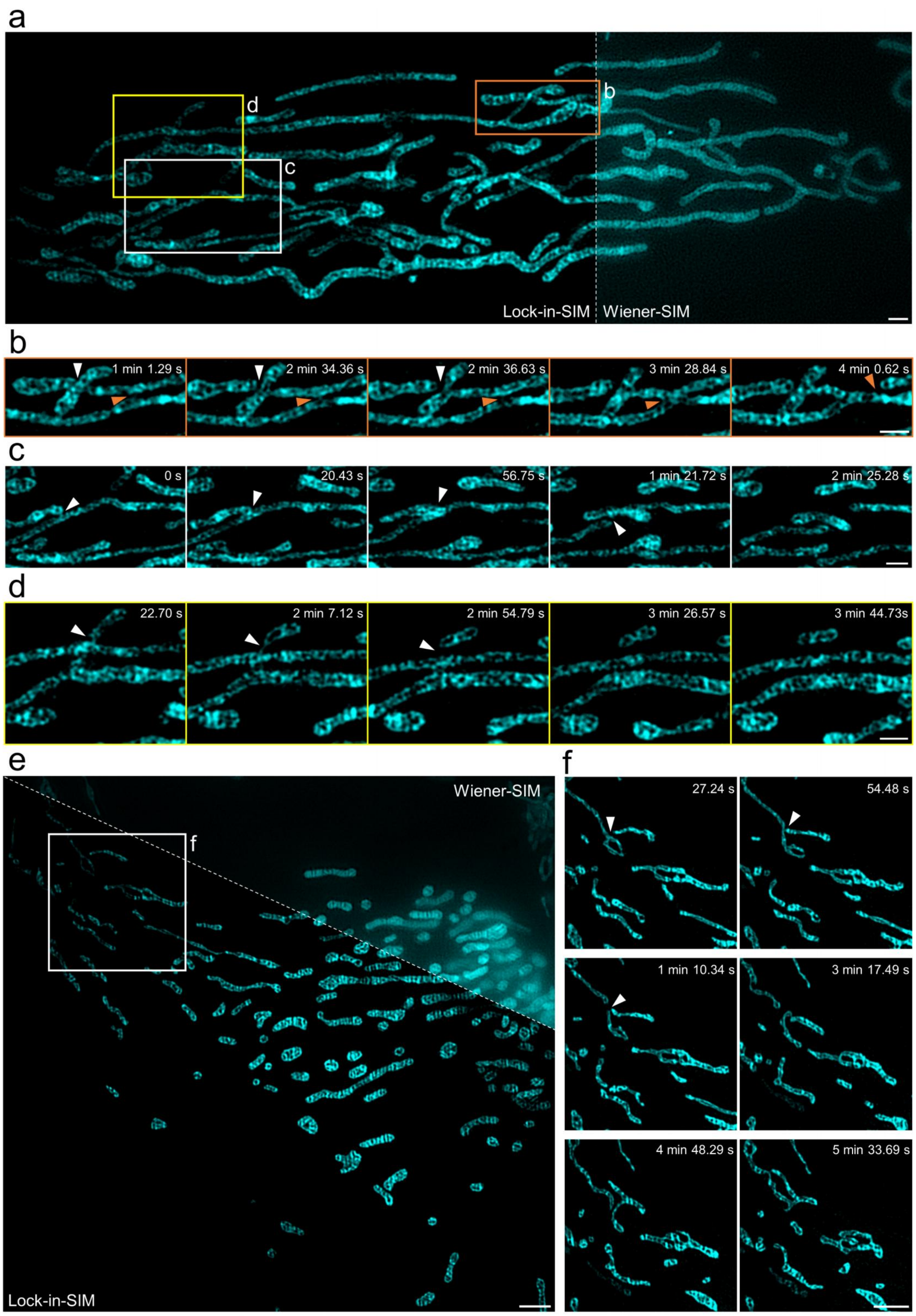

Supplementary Fig. 34. Live-cell Lock-in-SIM imaging of MMF. a, Lock-in-SIM (left) and Wiener-SIM (right) images of PKmito DEEP RED-labeled living mitochondrial inner membranes. **b-d**, Time-lapse images of the orange (**b**), white (**c**), and yellow (**d**) boxed regions in **a**. **e,** Lock-in-SIM (bottom left) and Wiener-SIM (top right) images of the inner membranes of PKmito DEEP RED-labeled living mitochondria. **f**, Time-lapse images of the white boxed region in **e**. All arrowheads indicate the MMF events. Scale bars, 1 μm (**a-d**) and 2 μm (**e,f**).

a

b

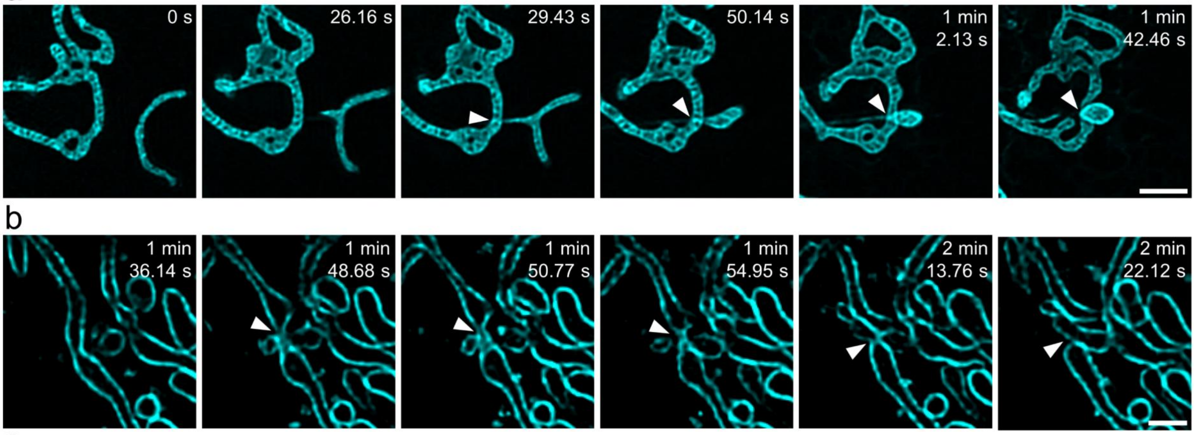

c

Mitochondrial fission	Probability
without another mitochondrion mediation	78.5% (51)
with another mitochondrion mediation (**MMF**)	21.5% (14)

Supplementary Fig. 35. Live-cell Lock-in-SIM imaging of MMF. a, b, Time-lapse images of the PKmito DEEP RED-labeled mitochondrial inner membranes (**a**) and Tomm20-EGFP-labeled mitochondrial outer membranes (**b**) showing one mitochondrion at the division site of another mitochondrion. White arrowheads indicate the MMF sites. Scale bars, 2 μm (**a**) and 1 μm (**b**). **c**, Quantitative statistics of the MMF probability among all mitochondrial fission events (n = 65, 8 cells).

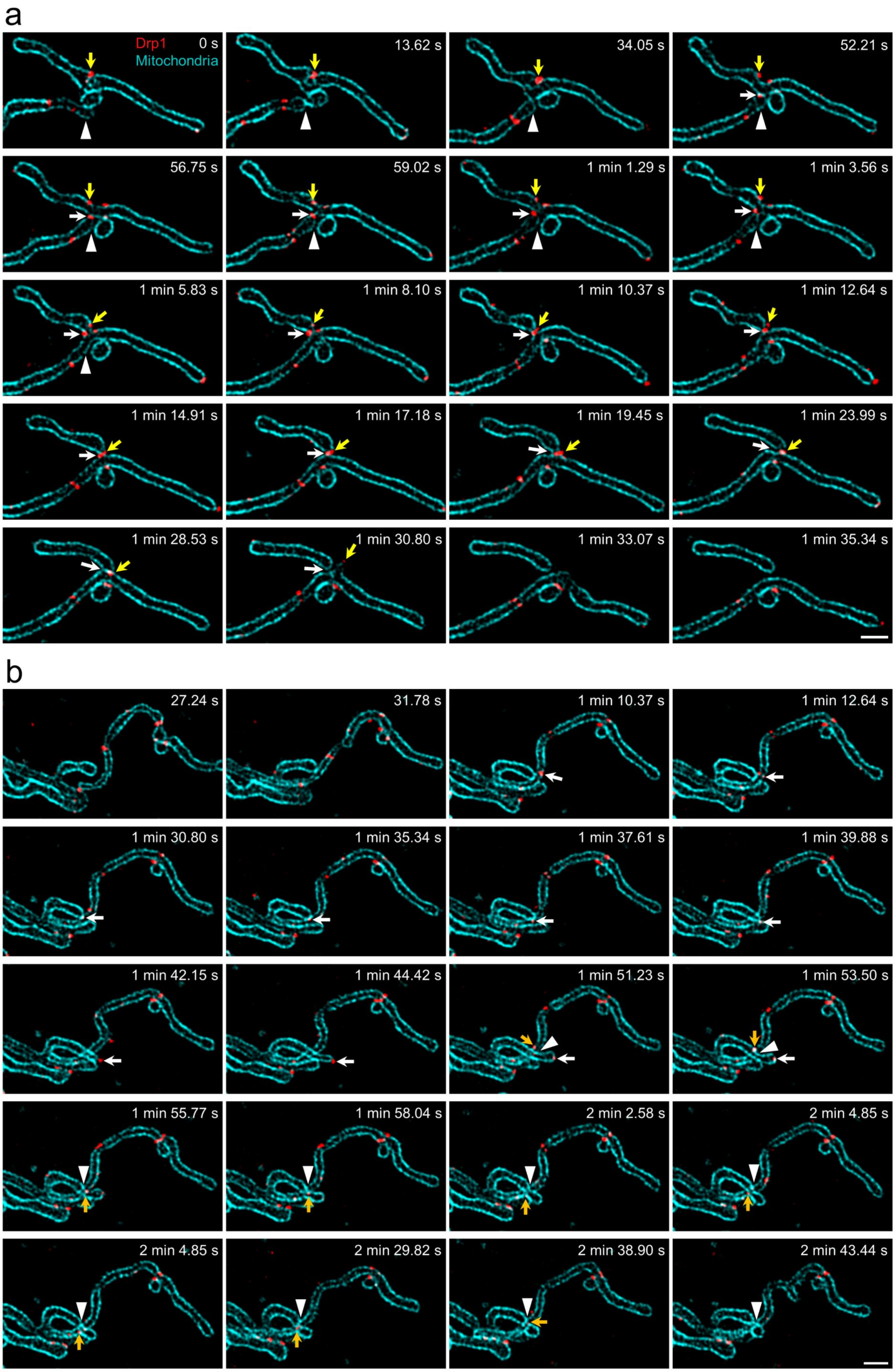
a
Drp1
Mitochondria
0 s
13.62 s
34.05 s
52.21 s
56.75 s
59.02 s
1 min 1.29 s
1 min 3.56 s
1 min 5.83 s
1 min 8.10 s
1 min 10.37 s
1 min 12.64 s
1 min 14.91 s
1 min 17.18 s
1 min 19.45 s
1 min 23.99 s
1 min 28.53 s
1 min 30.80 s
1 min 33.07 s
1 min 35.34 s
b
27.24 s
31.78 s
1 min 10.37 s
1 min 12.64 s
1 min 30.80 s
1 min 35.34 s
1 min 37.61 s
1 min 39.88 s
1 min 42.15 s
1 min 44.42 s
1 min 51.23 s
1 min 53.50 s
1 min 55.77 s
1 min 58.04 s
2 min 2.58 s
2 min 4.85 s
2 min 4.85 s
2 min 29.82 s
2 min 38.90 s
2 min 43.44 s

Supplementary Fig. 36. Two-color live-cell Lock-in-SIM imaging of MMF via the transport of Drp1. a, Two-color time-lapse images of Tomm20-EGFP-labeled mitochondrial outer membrane (cyan) and mCherry-labeled Drp1 oligomers (red). White arrowheads indicate the MMF event. Yellow arrows indicate the Drp1 oligomer on the fission mitochondria. White arrows indicate the Drp1 oligomer transported by the other mitochondria to participate in the mitochondrial fission. **b**, Two-color time-lapse images of the Tomm20-EGFP-labeled mitochondrial outer membrane (cyan) and mCherry-labeled Drp1 oligomers (red). White arrowheads indicate the MMF event. Orange arrows indicate the Drp1 oligomer transported by the other mitochondria to participate in the mitochondrial fission. White arrows indicate the transport of Drp1 oligomer between different mitochondria. Scale bars, 1 μm (**a,b**).

Supplementary Table 1. Qualitative comparison of Lock-in-SIM with other state-of-the-art 2D-SIM methods.

**Method**		**Performance**					**Open-access**		
		**Lateral resolution**	**SBR**	**Fidelity**	**2D imaging depth**	**3D Optical sectioning**	**MAT-LAB**	**Fiji/ ImageJ**	**Exe**
**Optical**	**TIRF-SIM**[13]	+ + +	+ + +	+ +	- -	-	/	/	/
	**GI-SIM**[14]	+ + +	+ + +	+ +	-	-	/	/	/
**Algorithm**	**OS-SIM**[5]	-	+ + +	+	+	+ + +	/	/	/
	**Wiener-SIM**[15]	+	+	+	+	+	/	/	/
	**WLR-SIM**[7]	+	+ +	+	+	+ +	No	No	No
	**Open-SIM**[16]	+	+	+	+	+	Yes	No	No
	**Fair-SIM**[17]	+	+	+	+	+	No	Yes	No
	**Hessian-SIM**[18]	+	+	+ +	+	+	Yes	No	Yes
	**IM-SIM**[4]	+	+	+	+	+	Yes	No	No
	**SP-SIM**[19]	+	+	+	+	+	Yes	No	No
	**HiFi-SIM**[1]	+	+ +	+ +	+	+ +	Yes	No	No
	**JSFR-SIM**[11]	+ +	+ +	+	+	+ +	Yes	No	No
	**PCA-SIM**[20]	+	+	+	+	+	Yes	No	No
	**BF-SIM**[9]	+ +	+ +	+ +	+	+ +	Yes	No	Yes
	**Direct-SIM**[10]	+ +	+ +	+	+	+ +	Yes	No	No
	**Flex-SIM**[12]	+ +	+ +	+ +	+	+ +	Yes	No	No
	**Lock-in-SIM**	**+ + +**[a]	**+ + +**	**+ + +**[b]	**+**	**+ + +**	**Yes**	**Yes**	**Yes**

Note: The benchmark is the classical Wiener-SIM, whose performance is denoted as “+”. More “+” signs mean higher performance, and more “-” signs mean lower performance.

[a] Although the lateral resolution of Lock-in-SIM is still limited by the SIM principle, its effective image resolution (i.e., image contrast) is enhanced because of the improved SBR.

[b] Lock-in-SIM can also be implemented as a pre-background filtering step to be combined with established SIM algorithms, including noise-suppressed algorithms, for subsequent super-resolution reconstruction under extremely low light levels.

Supplementary Table 2. Reconstruction parameters and speed of the algorithms.

	Parameters setting	Reconstruction speed (s)[a]
**WLR-SIM**	/[b]	
**Open-SIM**	No parameter setting	44.63
**Fair-SIM**	Wiener parameter=0.05, APO cutoff=2, APO bend=0.9	/[c]
**Hessian-SIM**	Wiener parameter=2, Theta Ratio=1:1:1, Mu=150, Sigma=1	/[d]
**IM-SIM**	wiener_factor=0.05	4.02
**SP-SIM**	wienerFactor=0.05	1.60
**HiFi-SIM**	attStrength=0.6-0.9, ApoFWHM=0.5, β=1, w1=1.2	9.23
**JSFR-SIM**	WIENER PARAMETER=0.5, AMP=1, SIGMA=1.5	/[c]
**PCA-SIM**	sub_optimization=1, Filter_size=11, Mask_size=3	3.79
**BF-SIM**	Camera Offset=100, Wiener para=1.5	44.88
**Direct-SIM**	α=0.8, β=0.5, AttStr.=0.3, FilterFWHM=0.5	5.23
**Flex-SIM**	params.OTFAttStr=0.999, params.OTFAttwdth=0.3, params.apodize = 0, params.sepOrr = 0, params.padSz=10, params.mu = 7e-6, params.regType=1, params.maxIt = 100, params.stepTol = 1e-3	43.32[e]
**Lock-in-SIM**	Lock-in parameter=0.5-0.8	8.68

[a] The speed was tested on a Windows 10 desktop equipped with a 13th Gen Intel(R) Core(TM) i5-13500 2.50 GHz CPU and 64 GB RAM, using a single-frame 512*512 2D-SIM raw dataset.

[b] The WLR-SIM reconstruction was performed and shared by the original authors.

[c] Reconstructed speeds for Fair-SIM and JSFR-SIM were not reported here due to their separated multi-step processing. Readers can refer to the original publications[11,17] for more details. Notably, JSFR-SIM is expected to offer highly increased speed due to the accelerated spatial-domain reconstruction.

[d] The reconstructed speed of Hessian-SIM was not reported here due to its multi-frame input requirement.

[e] The Flex-SIM speed was tested on a MacBook Pro laptop equipped with Apple M1 Pro and 16 GB RAM, due to the incomplete running on the Windows computer.

Supplementary References

1. Wen, G. et al. High-fidelity structured illumination microscopy by point-spread-function engineering. *Light-Sci. Appl.* **10**, 70 (2021).
2. He, Y. et al. Untrained neural network enhances the resolution of structured illumination microscopy under strong background and noise levels. *Adv. Photonics Nexus* **2**, 046005 (2023).
3. Qiao, C. et al. Rationalized deep learning super-resolution microscopy for sustained live imaging of rapid subcellular processes. *Nat. Biotechnol.* **41**, 367-377 (2023).
4. Cao, R. et al. Inverse matrix based phase estimation algorithm for structured illumination microscopy. *Biomed. Opt. Express* **9**, 5037-5051 (2018).
5. Saxena, M., Eluru, G. & Gorthi, S. S. Structured illumination microscopy. *Adv. Opt. Photonics* **7**, 241-275 (2015).
6. Marriott, G. et al. Optical lock-in detection imaging microscopy for contrast-enhanced imaging in living cells. *Proc. Nat. Acad. Sci. USA.* **105**, 17789-17794 (2008).
7. O'Holleran, K. & Shaw, M. Optimized approaches for optical sectioning and resolution enhancement in 2D structured illumination microscopy. *Biomed. Opt. Express* **5**, 2580-2590 (2014).
8. Dan, D. et al. Super-resolution and optical sectioning integrated structured illumination microscopy. *Journal of Physics D: Applied Physics* **54**, 074004 (2020).
9. Mo, Y. et al. Quantitative structured illumination microscopy via a physical model-based background filtering algorithm reveals actin dynamics. *Nat. Commun.* **14**, 3089 (2023).
10. Wen, G. et al. Spectrum-optimized direct image reconstruction of super-resolution structured illumination microscopy. *PhotoniX* **4**, 19 (2023).
11. Wang, Z. et al. High-speed image reconstruction for optically sectioned, super-resolution structured illumination microscopy. *Adv. Photonics* **4**, 026003-026003 (2022).
12. Soubies, E. et al. Surpassing light inhomogeneities in structured-illumination microscopy with FlexSIM. *J. Microsc.* **296**, 94-106 (2024).
13. Kner, P., Chhun, B. B., Griffis, E. R., Winoto, L. & Gustafsson, M. G. Super-resolution video microscopy of live cells by structured illumination. *Nat. Methods* **6**, 339-342 (2009).
14. Guo, Y. et al. Visualizing intracellular organelle and cytoskeletal interactions at nanoscale resolution on millisecond timescales. *Cell* **175**, 1430-1442 (2018).
15. Gustafsson, M. G. Surpassing the lateral resolution limit by a factor of two using structured illumination microscopy. *J. Microsc.* **198**, 82-87 (2000).
16. Lal, A., Shan, C. & Xi, P. Structured illumination microscopy image reconstruction algorithm. *IEEE Journal of Selected Topics in Quantum Electronics* **22**, 50-63 (2016).
17. Müller, M., Mönkemöller, V., Hennig, S., Hübner, W. & Huser, T. Open-source image reconstruction of super-resolution structured illumination microscopy data in ImageJ. *Nat. Commun.* **7**, 10980 (2016).
18. Huang, X. et al. Fast, long-term, super-resolution imaging with Hessian structured illumination microscopy. *Nat. Biotechnol.* **36**, 451-459 (2018).
19. Tu, S. et al. Fast reconstruction algorithm for structured illumination microscopy. *Opt. Lett.* **45**, 1567-1570 (2020).
20. Qian, J. et al. Structured illumination microscopy based on principal component analysis. *eLight* **3**, 4 (2023).
21. Qiao, C. et al. Evaluation and development of deep neural networks for image super-resolution in optical microscopy. *Nat. Methods* **18**, 194-202 (2021).
