# Supplementary material for: Visualizing intraorganellar ultrastructures, dynamics, and interactions with open-access background-free Lock-in-SIM
Source: Nat Commun. 2025 Nov 28;16:10765. doi: 10.1038/s41467-025-65805-w (PMC12663579; doi:10.1038/s41467-025-65805-w)
Supplement: Supplementary file 2 — Description of Additional Supplementary Files [file 41467_2025_65805_MOESM2_ESM.pdf]

Description of Additional Supplementary Files

Supplementary Movie 1: Optical sectioning and 3D rotating reconstruction comparison of simulated 3D filament structure without and with noise.

Supplementary Movie 2: 2D reconstruction comparison of microtubules using different 2D-SIM methods.

Supplementary Movie 3: Depth-coded max-intensity-projection (MIP) reconstruction comparison of 3D microtubules and mitochondria using different 2D-SIM methods.

Supplementary Movie 4: Depth-coded MIP reconstruction comparison of two-color 3D microtubules and lysosomes using different 2D-SIM methods.

Supplementary Movie 5: Optical sectioning and 3D rotating reconstruction comparison of 3D microtubules using different 2D-SIM methods.

Supplementary Movie 6: Optical sectioning and 3D rotating reconstruction comparison of 3D mitochondria using different 2D-SIM methods.

Supplementary Movie 7: Optical sectioning reconstruction comparison of 3D mitochondria (region of interest) using different 2D-SIM methods.

Supplementary Movie 8: Optical sectioning and 3D rotating reconstruction comparison of 3D microtubules using Lock-in-SIM and 3D-SIM methods.

Supplementary Movie 9: Live-cell imaging of microtubules.

Supplementary Movie 10: Live-cell two-color imaging of the ER and lysosomes, including lysosome tubule-directed lysosome moving, and ER tubule and matrix dynamics.

Supplementary Movie 11: Live-cell two-color imaging of ER-lysosome interactions, including ER-mediated lysosome tubule fission, lysosome movement in ER matrix, lysosome tubule-directed lysosome moving,

and ER matrix dynamics.

Supplementary Movie 12: Live-cell imaging of mitochondrial and inner membrane dynamics. Scale bar, 1 μm.

Supplementary Movie 13: Live-cell imaging of mitochondrial dynamics, including mitochondria-mediated mitochondrial fission (MMF), membrane connections between mitochondria, mitochondrial fusion and mitochondrial inner membrane dynamics.

Supplementary Movie 14: Live-cell two-color imaging of MMF (cyan) via the transport of Drp1 (red). Scale bar, 1 μm.
