# Supplementary material for: Visualizing intraorganellar ultrastructures, dynamics, and interactions with open-access background-free Lock-in-SIM
Source: Nat Commun. 2025 Nov 28;16:10765. doi: 10.1038/s41467-025-65805-w (PMC12663579; doi:10.1038/s41467-025-65805-w)
Supplement: Supplementary file 17 — Reporting Summary [file 41467_2025_65805_MOESM17_ESM.pdf]

nature portfolio

Corresponding author(s): Wenjie Liu, Lothar Schermelleh, Yu-Hui Zhang, Youhua Chen

Last updated by author(s): Sep 7, 2025

Reporting Summary

Nature Portfolio wishes to improve the reproducibility of the work that we publish. This form provides structure for consistency and transparency in reporting. For further information on Nature Portfolio policies, see our Editorial Policies and the Editorial Policy Checklist.

Statistics

For all statistical analyses, confirm that the following items are present in the figure legend, table legend, main text, or Methods section.

n/a	Confirmed	
☐	☒	The exact sample size (*n*) for each experimental group/condition, given as a discrete number and unit of measurement
☐	☒	A statement on whether measurements were taken from distinct samples or whether the same sample was measured repeatedly
☒	☐	The statistical test(s) used AND whether they are one- or two-sided<br>*Only common tests should be described solely by name; describe more complex techniques in the Methods section.*
☒	☐	A description of all covariates tested
☒	☐	A description of any assumptions or corrections, such as tests of normality and adjustment for multiple comparisons
☐	☒	A full description of the statistical parameters including central tendency (e.g. means) or other basic estimates (e.g. regression coefficient) AND variation (e.g. standard deviation) or associated estimates of uncertainty (e.g. confidence intervals)
☒	☐	For null hypothesis testing, the test statistic (e.g. *F*, *t*, *r*) with confidence intervals, effect sizes, degrees of freedom and *P* value noted<br>*Give P values as exact values whenever suitable.*
☒	☐	For Bayesian analysis, information on the choice of priors and Markov chain Monte Carlo settings
☒	☐	For hierarchical and complex designs, identification of the appropriate level for tests and full reporting of outcomes
☒	☐	Estimates of effect sizes (e.g. Cohen's *d*, Pearson's *r*), indicating how they were calculated

Our web collection on statistics for biologists contains articles on many of the points above.

Software and code

Policy information about availability of computer code

Data collection	The SIM datasets were mainly acquired using a custom-built high-speed SIM system equipped with 2D-SIM, TIRF-SIM, and 3D-SIM imaging modalities, and commercial SIM systems (GE DeltaVision OMX SR; CSR Biotech, HIS-SIM; and NanoInsights-Tec, Multi-SIM).
Data analysis	Fair-SIM reconstructions were performed using open-access Fair-SIM Fiji/ImageJ plugin (https://github.com/fairSIM/fairSIM). WLR-SIM reconstructions were performed by the original authors. Open-SIM (https://github.com/LanMai/OpenSIM), Hessian-SIM (https://www.nature.com/articles/nbt.4115), IM-SIM (https://github.com/rzcao/inverse_matrix_phase_algorithm/tree/v1.0.1), SP-SIM (https://github.com/hittsj/shiftPhaseSIM), HiFi-SIM (https://www.nature.com/articles/s41377-021-00513-w#Sec15), JSFR-SIM (https://codeocean.com/capsule/8064732/tree/v1), PCA-SIM (https://elight.springeropen.com/articles/10.1186/s43593-022-00035-x#Sec15), BF-SIM (https://figshare.com/articles/software/custom-written_MATLAB_code_and_its_test_example_data_for_BF-SIM/22640974/1), Direct-SIM (https://opticapublishing.figshare.com/s/6b7daa9f15e01de4e952?file=37543549), and Flex-SIM (https://github.com/esoubies/FlexSIM) reconstructions were performed using correspondcing open-access MATLAB cocdes with their default or recommended parameters (Supplementary Table 2). Some of the reconstructed results from the existing methods with their own datasets were, where available, either directly picked from their open-access files or reproduced with the provided parameter-optimized codes, such as BF-SIM, Direct-SIM, and Flex-SIM.<br>The quantitative, pixel-wise orientation values (θ) of fibrous structures, such as microtubules and mitochondria, were calculated with a weighted vector summation algorithm (https://github.com/WenjieLab/Order-index). The Fourier domain evaluation was performed using the SIMcheck Fiji/ImageJ plugin (https://www.micron.ox.ac.uk/software/SIMcheck/c). 3D volumetric data were mainly rendered and visualized with the ImageJ Volume Viewer plugin and Imaris software. The custom MATLAB codes, GUI, Fiji/ImageJ plugin, and executable software of Lock-in-SIM are available at https://github.com/WenjieLab/Lock-in-SIM.

For manuscripts utilizing custom algorithms or software that are central to the research but not yet described in published literature, software must be made available to editors and reviewers. We strongly encourage code deposition in a community repository (e.g. GitHub). See the Nature Portfolio guidelines for submitting code & software for further information.

Data

Policy information about availability of data

All manuscripts must include a data availability statement. This statement should provide the following information, where applicable:

- Accession codes, unique identifiers, or web links for publicly available datasets
- A description of any restrictions on data availability
- For clinical datasets or third party data, please ensure that the statement adheres to our policy

The source data that support the findings of this study are publicly available at https://figshare.com/articles/figure/Raw_datasets/26130994.

Research involving human participants, their data, or biological material

Policy information about studies with human participants or human data. See also policy information about sex, gender (identity/presentation), and sexual orientation and race, ethnicity and racism.

Reporting on sex and gender	N/A
Reporting on race, ethnicity, or other socially relevant groupings	N/A
Population characteristics	N/A
Recruitment	N/A
Ethics oversight	N/A

Note that full information on the approval of the study protocol must also be provided in the manuscript.

Field-specific reporting

Please select the one below that is the best fit for your research. If you are not sure, read the appropriate sections before making your selection.

☒ Life sciences ☐ Behavioural & social sciences ☐ Ecological, evolutionary & environmental sciences

For a reference copy of the document with all sections, see nature.com/documents/nr-reporting-summary-flat.pdf

Life sciences study design

All studies must disclose on these points even when the disclosure is negative.

Sample size	The sample size (n) of each experiment is provided in the figure legends. Typically, at least 3 replications were performed to ensure reproducibility.
Data exclusions	No data were excluded from the analysis.
Replication	The results can be fully replicated. First, we provide the user-friendly open-access software, data, and user guide for general using and testing. Secondly, we have tested our software on a lot of sample types and numbers (including published and our own). It all worked well. Corresponding data have been shown in the manuscript. Thirdly, the biological findings were observed independently in more than 10 different samples.
Randomization	No allocation into experimental group were performed.
Blinding	Blinding was not relevant to this study as we did not perform assays that depend on the experimenter.

Behavioural & social sciences study design

All studies must disclose on these points even when the disclosure is negative.

Study description	
Research sample	
Sampling strategy	

Data collection

Timing

Data exclusions

Non-participation

Randomization

Ecological, evolutionary & environmental sciences study design

All studies must disclose on these points even when the disclosure is negative.

Study description

Research sample

Sampling strategy

Data collection

Timing and spatial scale

Data exclusions

Reproducibility

Randomization

Blinding

Did the study involve field work? ☐ Yes ☐ No

Field work, collection and transport

Field conditions

Location

Access & import/export

Disturbance

Reporting for specific materials, systems and methods

We require information from authors about some types of materials, experimental systems and methods used in many studies. Here, indicate whether each material, system or method listed is relevant to your study. If you are not sure if a list item applies to your research, read the appropriate section before selecting a response.

Materials & experimental systems

n/a	Involved in the study	
☐	☒	Antibodies
☐	☒	Eukaryotic cell lines
☒	☐	Palaeontology and archaeology
☒	☐	Animals and other organisms
☒	☐	Clinical data
☒	☐	Dual use research of concern
☒	☐	Plants

Methods

n/a	Involved in the study	
☒	☐	ChIP-seq
☒	☐	Flow cytometry
☒	☐	MRI-based neuroimaging

Antibodies

Antibodies used	Primary antibody: Rabbit anti-GFP, abcam (cat#ab290), was used 1:600 dilution; mouse anti-alpha tubulin, abcam (cat#ab7291), was used 1:500 dilution; Rabbit anti-Tomm20 abcam (cat#ab232589), was used 1:500 dilution; Rabbit anti-Rab7, abcam (cat#ab137029), was used 1:500 dilution<br>Secondary antibody: Alexa Fluor 488 conjugated goat anti mouse antibody, abcam (cat#ab150113), was used 1:600 dilution; Alexa Fluor 488 conjugated goat anti rabbit antibody, abcam (cat#ab150077), was used 1:600 dilution; Cy3 conjugated goat anti rabbit antibody, abcam (cat#ab6939), was used 1:800 dilution
Validation	All the antibodies used in this study were validated by the vendor abcam and have been extensively used in previous studies. These labels include rabbit anti-GFP, abcam (cat#ab290): Chen J. et al. Nature Communications (2022). https://www.abcam.cn/products/primary-antibodies/gfp-antibody-ab290.html<br>mouse anti-alpha tubulin, abcam (cat#ab7291): Huang Z. et al. Science Advances (2021).<br>https://www.abcam.cn/alpha-tubulin-antibody-dmla-loading-controlab7291.html<br>rabbit anti-Tomm20, abcam (cat#ab232589): Gulen MF et al. Nature (2023).<br>https://www.abcam.cn/products/primary-antibodies/tomm20-antibody-epr15581-54--bsa-and-azide-free-ab232589.html<br>rabbit anti-Rab7, abcam (cat#ab137029): Wolf B. et al. Immunology (2022).<br>https://www.abcam.cn/products/primary-antibodies/rab7-antibody-epr7589-ab137029.html<br>Alexa Fluor 488 conjugated goat anti mouse antibody, abcam(cat#ab150113): Tulpule A. et al. Cell (2021).<br>https://www.abcam.cn/goat-mouse-igg-hl-alexa-fluor-488-ab150113.html<br>Alexa Fluor 488 conjugated goat anti rabbit antibody, abcam(cat#ab150077): Zhang c. et al. Nature Communications (2021).<br>https://www.abcam.cn/goat-rabbit-igg-hl-alexa-fluor-488-ab150077.html<br>Cy3 conjugated goat anti rabbit antibody, abcam (cat#ab6939): Chen J. et al. Cell (2023), https://www.abcam.cn/products/secondary-antibodies/goat-rabbit-igg-hl-cy3--preadsorbed-ab6939.html

Eukaryotic cell lines

Policy information about cell lines and Sex and Gender in Research

Cell line source(s)	U2OS and Cos7 cells were purchased from Procell Life Science & Technology Co., Ltd. Mouse C127 epithelial cells were from American Type Culture Collection.
Authentication	All the cell lines were authenticated by Procell Life Science & Technology, GATTA Quant, or ATCC and were not specifically authenticated in our lab.
Mycoplasma contamination	Cell lines used in this study tested negative for mycoplasma contamination.
Commonly misidentified lines<br>(See ICLAC register)	There were no misidentified cell lines used in this study.

Palaeontology and Archaeology

Specimen provenance	
Specimen deposition	
Dating methods	

☐ Tick this box to confirm that the raw and calibrated dates are available in the paper or in Supplementary Information.

Ethics oversight	

Note that full information on the approval of the study protocol must also be provided in the manuscript.

Animals and other research organisms

Policy information about studies involving animals; ARRIVE guidelines recommended for reporting animal research, and Sex and Gender in Research

Laboratory animals	
Wild animals	
Reporting on sex	
Field-collected samples	
Ethics oversight	

Note that full information on the approval of the study protocol must also be provided in the manuscript.

Clinical data

Policy information about clinical studies

All manuscripts should comply with the ICMJE guidelines for publication of clinical research and a completed CONSORT checklist must be included with all submissions.

Clinical trial registration	
Study protocol	
Data collection	
Outcomes	

Dual use research of concern

Policy information about dual use research of concern

Hazards

Could the accidental, deliberate or reckless misuse of agents or technologies generated in the work, or the application of information presented in the manuscript, pose a threat to:

No	Yes	
☐	☐	Public health
☐	☐	National security
☐	☐	Crops and/or livestock
☐	☐	Ecosystems
☐	☐	Any other significant area

Experiments of concern

Does the work involve any of these experiments of concern:

No	Yes	
☐	☐	Demonstrate how to render a vaccine ineffective
☐	☐	Confer resistance to therapeutically useful antibiotics or antiviral agents
☐	☐	Enhance the virulence of a pathogen or render a nonpathogen virulent
☐	☐	Increase transmissibility of a pathogen
☐	☐	Alter the host range of a pathogen
☐	☐	Enable evasion of diagnostic/detection modalities
☐	☐	Enable the weaponization of a biological agent or toxin
☐	☐	Any other potentially harmful combination of experiments and agents

Plants

Seed stocks	N/A
Novel plant genotypes	N/A
Authentication	N/A

ChIP-seq

Data deposition

☐ Confirm that both raw and final processed data have been deposited in a public database such as GEO.

☐ Confirm that you have deposited or provided access to graph files (e.g. BED files) for the called peaks.

Data access links
May remain private before publication.

Files in database submission

Genome browser session
(e.g. UCSC)

Methodology

Replicates

Sequencing depth

Antibodies

Peak calling parameters

Data quality

Software

Flow Cytometry

Plots

Confirm that:

☐ The axis labels state the marker and fluorochrome used (e.g. CD4-FITC).

☐ The axis scales are clearly visible. Include numbers along axes only for bottom left plot of group (a 'group' is an analysis of identical markers).

☐ All plots are contour plots with outliers or pseudocolor plots.

☐ A numerical value for number of cells or percentage (with statistics) is provided.

Methodology

Sample preparation

Instrument

Software

Cell population abundance

Gating strategy

☐ Tick this box to confirm that a figure exemplifying the gating strategy is provided in the Supplementary Information.

Magnetic resonance imaging

Experimental design

Design type

Design specifications

Behavioral performance measures

Acquisition

Imaging type(s)

Field strength

Sequence & imaging parameters

Area of acquisition

Diffusion MRI ☐ Used ☐ Not used

Preprocessing

Preprocessing software

Normalization

Normalization template

Noise and artifact removal

Volume censoring

Statistical modeling & inference

Model type and settings

Effect(s) tested

Specify type of analysis: ☐ Whole brain ☐ ROI-based ☐ Both

Statistic type for inference

(See Eklund et al. 2016)

Correction

Models & analysis

n/a	Involved in the study
☐	☐ Functional and/or effective connectivity
☐	☐ Graph analysis
☐	☐ Multivariate modeling or predictive analysis

Functional and/or effective connectivity

Graph analysis

Multivariate modeling and predictive analysis
